# Dendrite-targeting interneurons control synaptic NMDA-receptor activation via nonlinear α5-GABA_A_ receptors

**DOI:** 10.1038/s41467-018-06004-8

**Published:** 2018-09-03

**Authors:** Jan M. Schulz, Frederic Knoflach, Maria-Clemencia Hernandez, Josef Bischofberger

**Affiliations:** 1 Department of Biomedicine, University of Basel, Pestalozzistr. 20, CH-4056 Basel, Switzerland; 2 Pharma Research and Early Development, Discovery Neuroscience Department, F. Hoffmann-La Roche Ltd, CH-4070 Basel, Switzerland

## Abstract

Dendrite-targeting GABAergic interneurons powerfully control postsynaptic integration, synaptic plasticity, and learning. However, the mechanisms underlying the efficient GABAergic control of dendritic electrogenesis are not well understood. Using subtype-selective blockers for GABA_A_ receptors, we show that dendrite-targeting somatostatin interneurons and NO-synthase-positive neurogliaform cells preferentially activate α5-subunit- containing GABA_A_ receptors (α5-GABA_A_Rs), generating slow inhibitory postsynaptic currents (IPSCs) in hippocampal CA1 pyramidal cells. By contrast, only negligible contribution of these receptors could be found in perisomatic IPSCs, generated by fast-spiking parvalbumin interneurons. Remarkably, α5-GABA_A_R-mediated IPSCs were strongly outward-rectifying generating 4-fold larger conductances above –50 mV than at rest. Experiments and modeling show that synaptic activation of these receptors can very effectively control voltage-dependent NMDA-receptor activation as well as Schaffer-collateral evoked burst firing in pyramidal cells. Taken together, nonlinear-rectifying α5-GABA_A_Rs with slow kinetics match functional NMDA-receptor properties and thereby mediate powerful control of dendritic postsynaptic integration and action potential firing by dendrite-targeting interneurons.

## Abstract (web summary)

Somatostatin+ (SOM+ ) GABAergic interneurons are known to fine-tune synaptic plasticity as they inhibit dendritic spikes and burst firing. Here, the authors show that both SOM+ and NOS+ interneurons preferentially recruit nonlinear outward-rectifying GABA(A)R with alpha5 subunit, and that this inhibition with slow gating kinetics matches voltage and time-dependent activation of synaptic NMDARs, thereby controlling the generation of dendritic NMDA spikes.

## Introduction

Dendrite-targeting GABAergic interneurons control signal integration in pyramidal-cell dendrites to improve specific formation and selective recruitment of neuronal cell assemblies^1–3^. For example, somatostatin (SOM)- expressing GABAergic interneurons in rodent motor cortex control precision motor learning by regulating synaptic plasticity in distal L5-pyramidal-cell dendrites^4^. Furthermore, pharmacogenetic activation of SOM cells in the primary somatosensory cortex controls synaptic plasticity and somatosensory learning^5^. Similarly, hippocampal SOM interneurons powerfully inhibit dendritic spikes and burst firing in CA1 pyramidal cells and modulate hippocampus-dependent learning^6–8^. However, the underlying mechanisms are poorly understood.

Apical dendrites of pyramidal cells integrate thousands of excitatory and inhibitory synaptic inputs via active dendritic electrogenesis. In CA1 pyramidal cells, it was shown that synchronous pairing of Schaffer-collateral stimulation with distal entorhinal inputs generates NMDA-receptor (NMDAR)-dependent dendritic plateau potentials and burst firing, which can induce long-lasting changes in synaptic strength^9^. Consequently, the generation of plateau potentials and burst firing during spatial exploration can induce the formation of new place fields in CA1 pyramidal cells^10^. Using optogenetic silencing, it was shown that PV-positive soma-targeting basket cells affect spike timing of CA1 pyramidal cells relative to extracellular theta oscillations without much change in firing frequency during spatial exploration. By contrast, silencing of dendrite-targeting SOM interneurons strongly increases NMDAR-dependent burst firing in pyramidal cells^6,7^. This indicates that dendrite-targeting interneurons can more powerfully control NMDAR activation and synaptic plasticity than PV interneurons. This is surprising, as SOM interneurons are believed to fire significantly less action potentials (APs) than PV interneurons^11–13^. Furthermore, on the synaptic level, PV-basket cells are known to generate large-amplitude and precisely timed IPSCs in CA1 pyramidal cells^14–16^. In contrast to PV interneurons, SOM cells generate small-amplitude IPSCs^17^. Therefore, it is largely unclear how dendritic GABAergic synapses exert their powerful control of dendritic NMDAR activation and burst firing.

Using interneuron-specific genetic mouse lines and Cre-dependent ChrR2 expression, we studied kinetic properties and subunit composition of synaptic GABA_A_ receptors (GABA_A_Rs) activated by SOM interneurons and NO-synthase (NOS)-expressing neurogliaform cells, targeting distal dendrites of CA1 pyramidal cells. Using new subtype-selective modulators of GABA_A_Rs and detailed computational modeling, we specifically investigate the control of synaptic NMDAR activation by these dendrite-targeting interneurons. We show for the first time that both types, SOM and NOS interneurons preferentially recruit nonlinear outward-rectifying GABA_A_ receptors containing α5-subunits (α5-GABA_A_R) with slow gating kinetics, which match voltage- and time-dependent activation of synaptic NMDARs.

## Results

### α5-containing GABA_A_Rs mediate synaptic inhibition onto apical dendrites

Slow dendritic inhibition onto CA1 pyramidal cells was suggested to depend on activation of α5-GABA_A_Rs^18–21^. However, synaptic localization of these receptors has remained controversial^22–24^. While some studies using light microscopy indicated homogeneously distributed expression of α5-GABA_A_Rs along the (extrasynaptic) somatodendritic membrane of CA1 pyramidal cells^25,26^, others have suggested that these receptors are preferentially expressed in pyramidal-cell dendrites^23,27^. Furthermore, ultrastructural data suggested a specific enrichment in the postsynaptic membrane of hippocampal and neocortical pyramidal cells^23^.

**Fig. 1:**
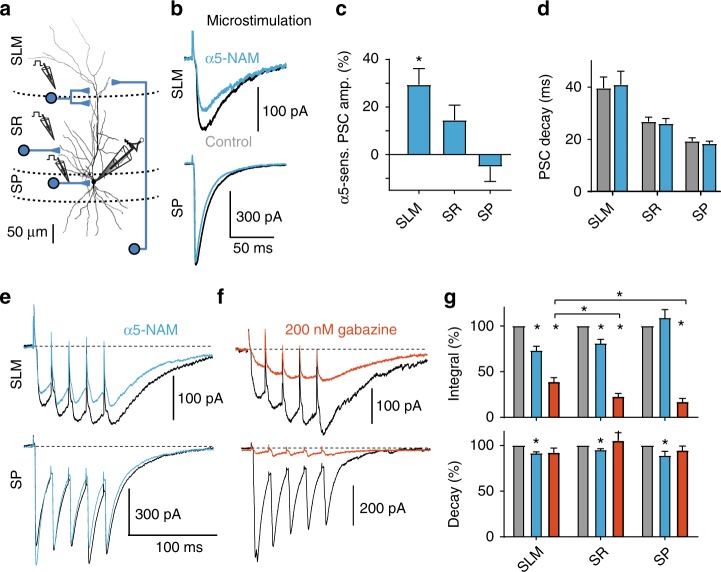
GABAergic synapses onto pyramidal-cell dendrites activate α5-GABA_A_Rs. **a** Experimental design. Targeted projections from local interneurons are indicated. **b** IPSCs evoked by electrical stimulation in different layers (SLM stratum lacunosum moleculare, SR stratum radiatum, SP stratum pyramidale) were recorded at –70 mV in the presence of 10 µM NBQX and 25 µM AP5 using a CsCl-based pipette solution. Representative mean IPSCs before (black) and after addition of the α5-NAM RO4938581 (1 µM, blue) show that only IPSCs evoked in the dendritic layers were sensitive to the α5-NAM. **c** Group means of the normalized α5-NAM-sensitive component in SLM-evoked IPSCs (*P* < 0.01; one sample *t* test, *n* = 6) and SR-evoked IPSCs (*P* = 0.085; Wilcoxon signed rank test, *n* = 11). **d** The mean decay time constant of single IPSCs evoked in different layers of CA1 was not affected by the application of the α5-NAM. **e** Brief-burst stimulation (5@50 Hz) of putative dendritic (SLM) and perisomatic (SP) IPSCs before and after (blue) application of the α5-NAM (1 µM, blue). **f** Effect of 200 nM gabazine on burst IPSCs. **g** Top, bar graph showing that application of the α5-NAM reduced the IPSC burst integral of SLM- (*P* < 0.01; paired *t* test, *n* = 6) and SR-evoked IPSCs (*P* < 0.01; *n* = 11), whereas somatic IPSCs evoked in SP remained unaffected (*P* = 0.35; *n* = 10). Conversely, gabazine-induced reductions (200 nM) were significantly smaller in SLM- than in SR- or SP-evoked IPSCs (both: *P* *<* 0.01; Mann–Whitney test). Bottom, the α5-NAM reduced the burst IPSC decay *τ* in all three cases (all: *P* < 0.05; paired *t* test). Recording temperature ≈ 33 °C

To test the contribution of α5-GABA_A_Rs to synaptic inhibition, we used RO4938581, a new highly selective negative allosteric modulator for α5-GABA_A_Rs^28,29^ (α5-NAM). Inhibitory postsynaptic currents (IPSCs) were recorded at resting membrane potential (–70 mV) with symmetrical Cl^−^ solutions after stimulation in different dendritic layers or close to the soma of CA1 pyramidal neurons in the presence of NBQX and AP5 (Fig. 1a). Stimulation in stratum lacunosum moleculare (SLM) evoked IPSCs with slow kinetics (Fig. 1b, top). These slow putative dendritic IPSCs were significantly reduced in amplitude by 29.4 ± 7.1% after the application of the α5-NAM (1 μM) from 138.8 ± 23.3 pA to 95.9 ± 14.6 (*n* = 6; *P* < 0.01, paired *t* test; Fig. 1c). In contrast, fast perisomatic IPSCs evoked in stratum pyramidale (SP) were not significantly decreased (534.5 ± 96.4 vs. 533.8 ± 107.6 pA, *P* = 0.98, *n* = 7; Fig. 1c, d). Stimulation in stratum radiatum (SR) evoked IPSCs of intermediate dynamics and intermediate α5-NAM sensitivity (Fig. 1c). The effect of RO4938581 was specific to GABAergic synapses, as there was no change in amplitude and time course of dendritic EPSCs evoked in the presence of 100 µM picrotoxin (Supplementary Figure 1). These results suggested that α5-GABA_A_R contributes to slow dendritic inhibition, while fast perisomatic inhibition was largely unaffected.

In order to understand the contribution of α5-GABA_A_R to inhibition during enhanced interneuron activity, we applied a brief-burst stimulation (5@50 Hz). Stimulation in SLM induced temporal summation of the slow IPSCs to a peak amplitude of 231.4 ± 45.2 pA (*n* = 6), which was reduced by 24.6 ± 5.1% after addition of the α5-NAM (*P* < 0.01, paired *t* test). Similarly, the area under the curve of the burst PSC was substantially reduced by 26.7 ± 5.2% (*P* < 0.01; Fig. 1e–g). Although RO4938581 is very selective for α5-GABA_A_R, the maximal efficacy of this modulator is only ~50%^28^. Taking into account the intrinsic efficacy of the compound, the contribution of these GABA_A_Rs is twice as large as the reduced current amplitude, suggesting that about 50% of the IPSC in SLM is generated by α5-GABA_A_R. In contrast to IPSCs evoked at dendritic layers, IPSCs evoked by burst stimulation in SP were not significantly reduced by the α5-NAM (*P* = 0.35, *n* = 10; Fig. 1e–g). Similarly, spontaneous IPSCs had rapid rise and decay kinetics and were largely unaffected by the α5-NAM (Supplementary Figure 2). Conversely, somatic IPSCs evoked in SP were fully blocked by application of low concentrations of gabazine (200 nM), while IPSCs evoked in SLM were only reduced to 38.7 ± 4.8% of control (*n* = 11, *P* = 0.001, Wilcoxon signed rank test; Fig. 1f, g). Apparently, the slow α5-GABA_A_R-mediated IPSCs are much less sensitive to gabazine than the somatic non-α5-mediated IPSCs, consistent with a higher affinity for GABA in α5-subunit-containing receptors. These results indicate that high-affinity α5-GABA_A_R significantly contributes to GABAergic synapses in CA1 pyramidal-cell dendrites, but shows negligible contribution to perisomatic IPSCs.

Interestingly, the application of the α5-NAM resulted in a small decrease of the burst IPSC decay time constant independent of stimulation site (Fig. 1g, bottom) supporting the idea that perisynaptic α5-GABA_A_Rs may contribute to inhibition during synaptic burst activity. Extrasynaptic α5-GABA_A_Rs have also been implicated in tonic inhibition^30^. Therefore, we examined the relative contribution of α5-GABA_A_Rs to tonic inhibition (Supplementary Figure 2G). In the presence of 5 µM GABA, the application of the α5-NAM RO4938581 reduced the GABA-dependent tonic currents by 37.7 ± 5.5% (*n* = 11), consistent with 75% of tonic inhibition in CA1 pyramidal neurons mediated via α5-GABA_A_Rs in line with previous reports^31^.

Taken together, these results suggest that GABAergic synapses targeting CA1 pyramidal-cell dendrites recruit high-affinity α5-containing GABA_A_Rs, contributing to about 50% of the IPSC, in strong contrast to perisomatic inhibition.

### Reduction of α5-GABA_A_R-mediated dendritic inhibition facilitates activation of NMDARs

**Fig. 2:**
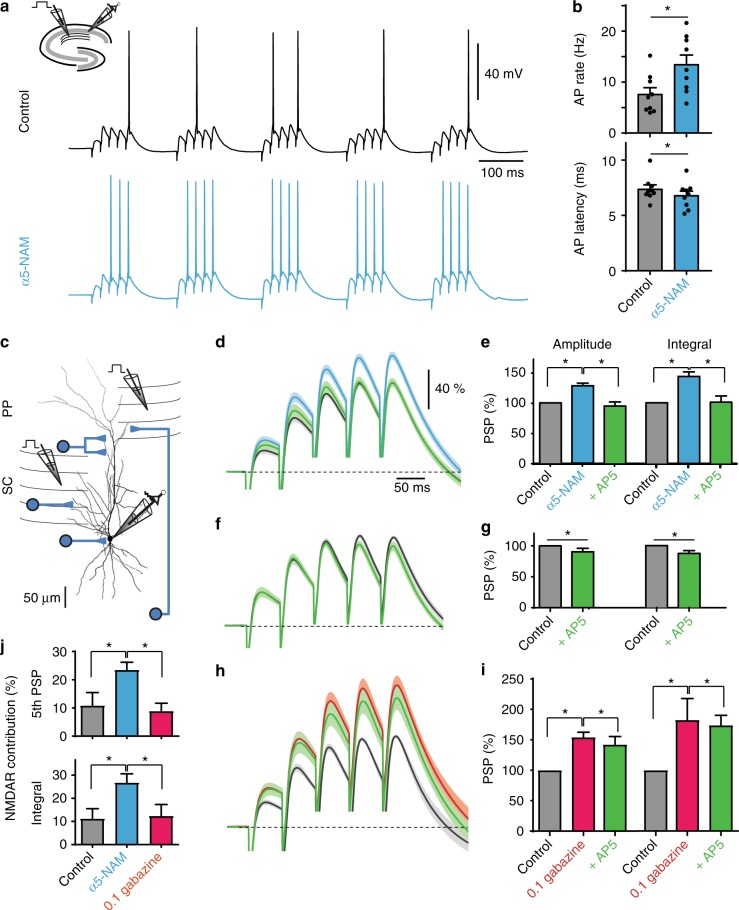
Activation of α5-GABA_A_Rs controls dynamic NMDAR recruitment. **a** Example voltage traces of theta-burst evoked AP firing during distant Schaffer-collateral (SC) stimulation before and after the addition of the α5-NAM RO4938581 (1 µM). Inset shows the experimental design. **b** Group means show a significantly increased spike rate (*P* < 0.05; paired *t* test, *n* = 9) and decreased latency between stimulation and AP discharge (*P* < 0.05; *n* = 9). **c** Experimental design for local stimulation of glutamatergic inputs from Schaffer collaterals (SC) and perforant path (PP) as well as associated GABAergic inputs. **d** Enhanced contribution of NMDARs after application of the α5-NAM. The grand means with SEM (lighter shades) of 12 experiments are shown. Each mean was normalized to the maximal voltage deflection in the control condition (gray). Application of the α5-NAM (1 µM) increased the burst PSP (blue). The addition of AP5 (50 µM, green) completely reversed this effect. **e** Group means of the amplitude and integral of the burst PSP. Statistical significant differences are indicated (*P* < 0.001; paired *t* test, *n* = 12). **f**, **g** Application of AP5 (50 µM, green) in the absence of α5-NAM caused much smaller changes in amplitude and integral (*P* < 0.05; paired t test, *n* = 8). **h**, **i** Application of low concentration of gabazine (0.1 µM, red) increased amplitude and integral to a comparable degree as α5-NAM (*P* < 0.01; paired *t* test, *n* = 8). Yet the relative effect of subsequent AP5 application was much weaker. **j** The relative contribution of NMDAR-mediated depolarization was significantly higher after the addition of the α5-NAM than under control conditions or after the addition of 0.1 µM gabazine (all: *P* < 0.05; two-sample *t* test). *T* ≈ 33 °C

What is the functional role of synaptic α5-GABA_A_Rs in CA1 pyramidal cells? To test the effect of α5-GABA_A_Rs on dendritic integration, we performed whole-cell current-clamp recordings in the absence of any blockers and stimulated Schaffer-collateral inputs using a theta-burst paradigm (five bursts every 200 ms with 5@50 Hz each, Fig. 2a). Application of the α5-NAM strongly increased AP rate (13.5 ± 1.8 vs. 7.6 ± 1.3 Hz, *P* < 0.05; paired *t* test, *n* = 9; Fig. 2b) and decreased AP latency within the burst (6.8 ± 1.2 vs. 7.4 ± 1.1 ms, *P* < 0.05; *n* = 9). These results show that α5-GABA_A_Rs can powerfully regulate dendritic integration of synaptic inputs as well as its conversion into AP firing.

As extrasynaptic α5-GABA_A_Rs have been implicated in tonic inhibition, we asked whether the observed effects could be explained by changes in tonic inhibition induced by the α5-NAM. Therefore, we analyzed the effect of α5-NAM (1 µM) on passive membrane properties (Supplementary Figure 3). Application of α5-NAM increased the input resistance (*R*_in_) by 3.9 ± 1.5% (*P* < 0.05, *n* = 18), leading to a small decrease of the current threshold for AP generation by 11 ± 4 pA (*P* < 0.05, *n* = 6). This small change in *R*_in_ via extrasynaptic receptors is unlikely to account for the dramatic effect of α5-NAM on AP output and suggested that synaptic α5-GABA_A_Rs might substantially contribute to this effect.

As synaptic α5-GABA_A_R-mediated currents show slow kinetics similar to NMDAR currents, GABAergic synapses could potentially interfere with NMDAR activation^32^. To test the impact of synaptic α5-GABA_A_Rs on the activation of NMDARs, we stimulated synaptic inputs in SR and SLM with low intensity and recorded subthreshold burst PSPs with an average amplitude of 10.8 ± 0.6 mV (*n* = 12; Fig. 2c). After the addition of the α5-NAM (1 μM), the burst amplitude and integral increased to 131.9 ± 3.4% and 147.9 ± 5.3% (*n* = 12) of control, respectively (Fig. 2d, e, blue). Subsequent addition of AP5 (50 µM) decreased the amplitude and integral back to 102.3 ± 4.2% and 108.7 ± 7.4% of control levels, respectively (*P* < 0.001, paired *t* test; Fig. 2d, e, green). By contrast, application of AP5 in ACSF caused only a small change in the PSP amplitude (90.9 ± 3.1%, *P* < 0.05, *n* = 8) and integral (89.0 ± 4.5%, *P* < 0.05; Fig. 2f, g). Similar results were obtained using the well-known α5-GABA_A_R-selective inverse agonist (α5-IA) L-655,708^20,33^ (Supplementary Figure 4). These results demonstrate that activation of synaptic α5-GABA_A_Rs effectively decreases NMDAR-mediated dendritic depolarization in CA1 pyramidal cells.

As reduced inhibition is expected to increase EPSP amplitudes and eventually activation of NMDARs, the results above may not be specific to α5-GABA_A_Rs. Thus, we aimed to compare the relative effects of negative modulation of α5-GABA_A_Rs with a preferential block of non-α5-receptor-mediated inhibition via a low concentration of gabazine. Application of gabazine (0.1 µM) increased the amplitude and integral of burst PSPs to a comparable extent as the α5-NAM to 153.5 ± 10.3% and 195.8 ± 16.7% (*n* = 8) of control, respectively (Fig. 2h, i). However, subsequent application of AP5 had a relatively weak effect. In fact, the contribution of NMDARs to the PSP in gabazine was similar to the one in control condition and significantly smaller than in the presence of the α5-NAM (Fig. 2j).

These results show that low concentrations of gabazine equally affect both subthreshold NMDA- and non-NMDA-receptor-mediated PSPs, while α5-GABA_A_Rs preferentially control NMDA-mediated depolarization. Apparently, a 25% reduction of dendritic inhibition mediated via α5-GABA_A_Rs is sufficient to generate an NMDA-dependent 1.5-fold increase in subthreshold EPSP and a 2-fold increase in spiking output. Therefore, dendrite-targeting interneurons activating synaptic α5-GABA_A_Rs will be able to preferentially control NMDA-dependent spiking and plasticity.

### SOM and NOS interneurons target synaptic α5-GABA_A_Rs on pyramidal-cell dendrites

The identity of the GABAergic interneurons activating α5-GABA_A_Rs in CA1 pyramidal-cell dendrites is unknown. Among the several classes of interneurons targeting the apical dendritic tree of CA1 pyramidal neurons, somatostatin (SOM)-expressing stratum oriens-lacunosum moleculare (O-LM) interneurons and NOS-expressing neurogliaform cells are prototypical dendrite-targeting interneurons. Furthermore, their synapses dominate the distal regions in SLM^11,34,35^. Therefore, we focused on SOM- and NOS-expressing interneurons and compared the functional properties of their GABAergic synapses with properties of parvalbumin (PV)-interneuron-mediated perisomatic inhibition.

**Fig. 3:**
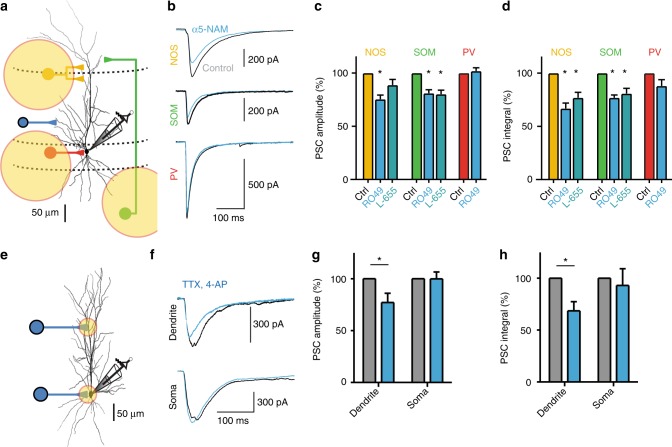
SOM and NOS interneurons mediate dendritic inhibition via α5-GABA_A_Rs. **a** Experimental design. Optogenetically targeted projections from local interneurons and the respective fields of illumination. **b** Light-evoked IPSCs from different interneuron populations (NOS, yellow; SOM, green; PV, red) were recorded at –70 mV in the presence of 10 µM NBQX and 25 µM AP5 using a CsCl-based pipette solution. Representative mean IPSCs before and after addition of the α5-NAM RO4938581 (1 µM, blue) show that IPSCs evoked in dendrite-targeting interneurons (NOS and SOM) were reduced. **c** Group means of the normalized IPSC amplitude showed a significant reduction of NOS (*P* < 0.001; paired *t* test, *n* = 8) and SOM-mediated IPSCs (*P* < 0.01; *n* = 7) after the addition of RO4938581, while PV-mediated IPSCs were not affected (*P* = 0.57, *n* = 8; paired *t* test). In addition, the effect of the α5-IA L-655,708 (50 nM) on the amplitude of SOM (*P* < 0.01, *n* = 6) and NOS IPSCs is shown (*P* = 0.07, *n* = 7). **d** The mean normalized PSC integral was reduced after the application of both α5-GABAR modulators for inputs from NOS and SOM interneurons (all: *P* < 0.05; paired *t* test, *n* = 8), while PV synapses were not affected (*P* = 0.08, *n* = 8; paired *t* test). **e** Schematic of ChR2-assisted input mapping. **f** Responses to stimulation of dendritic (200–300 µm from soma, top) and somatic inputs (bottom) before and after addition (blue) of the α5-NAM. **g**, **h** Group means show a significant reduction in PSC amplitude and integral for dendritic inputs (*P* < 0.05; paired *t* test, *n* = 8), but not for perisomatic synapses (*P* > 0.6; *n* = 8). Recording temperature *T* ≈ 22 °C

NOS interneurons were selectively activated using nNOS-Cre X flox-ChR2 mice (NOS-ChR2) and a brief 5-ms pulse of laser light was applied to SLM (473 nm, Fig. 3a). Light-evoked IPSCs showed slow kinetics and an α5-NAM-sensitive amplitude (24.6 ± 4.0%, *P* < 0.001, *n* = 8; Fig. 3b, c), similar to the SLM-evoked IPSCs. Furthermore, light-induced activation of SOM interneurons in stratum oriens (SO) of SOM-Cre X flox-ChR2 mice (SOM-ChR2) evoked slow IPSCs that were significantly reduced by 18.9 ± 3.7% during α5-NAM application (*P* < 0.01, *n* = 7; Fig. 3b, c). Similarly, the synaptic PSC integral was reduced after application of the α5-NAM by 33.2 ± 4.1% (*P* < 0.001) and 23.1 ± 1.9% (*P* < 0.001) in the NOS- and SOM-ChR2-evoked responses, respectively (Fig. 3d). By contrast, the amplitude and integral of fast IPSCs evoked by light stimulation in SP of PV-Cre X flox-ChR2 mice (PV-ChR2), were not affected by the α5-NAM (*P* = 0.57 and *P* = 0.08, *n* = 8). Finally, application of the classical α5-IA L-655,708 (50 nM) reduced light-evoked currents in NOS-ChR2 (*n* = 7 cells) and SOM-ChR2 (*n* = 6 cells) animals to a similar extent as RO4938581 (Fig. 3c, d).

Taking into account the maximal efficacy of our α5-NAM, these results show that GABAergic synapses formed by NOS- and SOM-positive interneurons recruit synaptic α5-GABA_A_Rs, contributing to about 50–60% of their IPSCs at resting potential.

Electrical or optogenetic stimulation of axons could potentially activate remote GABAergic synapses via axonal projections. To rigorously test the hypothesis that postsynaptic α5-GABA_A_Rs are located at dendritic synapses rather than in perisomatic synapses, we studied localized synaptic GABA release using subcellular channelrhodopsin-assisted circuit mapping^36,37^ (sCRACM). Targeted laser light stimulation was used to evoke GABA release in brain slices from mice expressing channelrhodopsin-2 under the control of the VGAT promoter (VGAT-Chr2, Fig. 3e). As axonal conduction was blocked by the addition of TTX (1 μM), the activation of GABA synapses was spatially restricted to the field of illumination^36^ (diameter of 20–50 μm). After the addition of the α5-NAM, light-evoked IPSCs in the apical dendrite at 200–300 µm from the soma were decreased in amplitude and integral to 77.1 ± 8.9% (*P* < 0.05) and 68.5 ± 8.9% (*P* < 0.01; *n* = 8), respectively (Fig. 3f–h). By contrast, the amplitude of light-activated perisomatic IPSCs remained unchanged (*P* = 0.99; *n* = 8). These results firmly established that α5-GABA_A_Rs selectively mediate slow GABAergic inhibition targeting CA1 pyramidal-cell dendrites.

### SOM- and NOS-interneuron-induced IPSCs show strong outward rectification

**Fig. 4:**
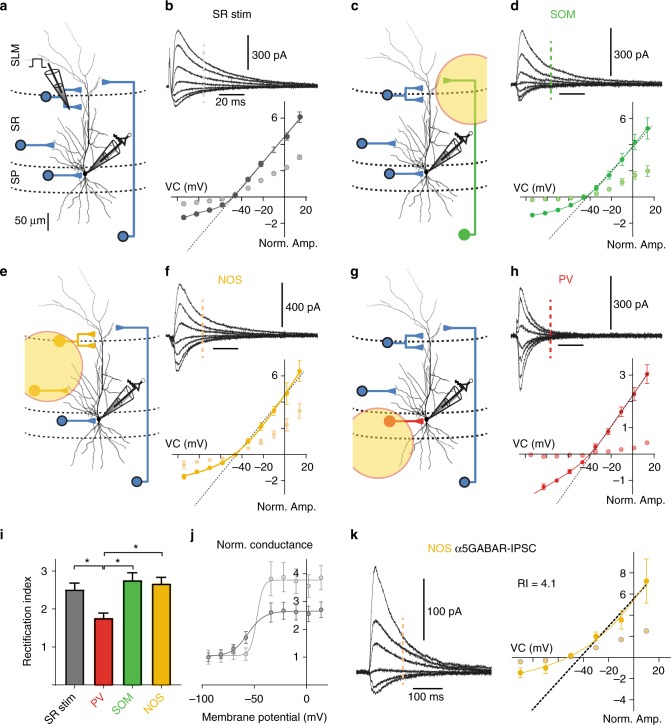
Dendritic synaptic inhibition by SOM and NOS interneurons is strongly outward rectifying. **a** Experimental design for local stimulation of GABAergic inputs in the outer third of SR. **b** IPSCs evoked at the border between SLM and SR were recorded at different membrane potentials in the presence of 10 µM NBQX and 25 µM AP5 using a Cs-gluconate-based solution. Bottom, group data (*n* = 11) show the mean IPSC amplitudes depending on the holding potential. Dark gray depicts the peak values, light gray the amplitude of the tail current measured at 30 ms after stimulation (indicated by dashed line in the top panel). The solid line represents a nonlinear fit according to equation 1 (Methods), while the dashed line represents a linear fit to values above –40 mV. **c**–**h** The same for optogenetically activated inputs from specific interneuron populations. Field of illumination relative to the recorded cell is indicated. **i** Mean rectification indices (RI) were quantified as the ratio of the conductance extrapolated from the linear fit to the outward currents above –40 mV divided by the measured value at –82 mV (1 = no deviation). The mean RI in PV-ChR2 stimulations (*n* = 8) was significantly smaller than in all experiments with dendritic inputs (SR: *P* < 0.01; paired *t* test, *n* = 11; NOS: *P* < 0.001, *n* = 9; SOM: *P* < 0.001, *n* = 9). **j** The voltage dependence of the peak (dark gray) and tail conductance (light gray) for electrically stimulated dendritic inputs. Conductances were normalized to –82 mV. **k** The grand mean of α5-GABAR-mediated IPSCs from NOS-ChR-stimulated IPSCs (*n* = 10) isolated by application of L-655,708 (50 nM). Mean IPSC amplitude is plotted against holding potential. Tail currents measured at 120 ms after stimulation are indicated (lighter shaded dots). The RI was calculated as the ratio of the conductance extrapolated from the linear fit to the outward currents above –40 mV (dashed line) divided by the conductance at –82 mV interpolated by the nonlinear fit to the measured PSCs (solid line). Recording temperature *T* ≈ 33 °C for **a** to **j**, and *T* ≈ 22 °C for **k**

Extrasynaptic GABA_A_Rs in CA1 pyramidal cells largely consist of α5-GABA_A_Rs and were reported to show outward rectification^38^. Furthermore, outward currents at depolarized potentials are especially relevant for inhibition of NMDAR-dependent dendritic plateau potentials by dendritic GABAergic inputs. Therefore, we examined the possibility that dendrite-targeting synaptic inhibition mediated by α5-GABA_A_Rs would also show outward rectification. During local stimulation in the distal SR, we recorded IPSCs at different holding potentials from –90 to + 20 mV (Fig. 4a). The pipette solution was Cs-gluc based and contained a physiologically low chloride concentration (8 mM). Under these conditions, peak amplitudes of outwardly directed IPSCs increased linearly with holding potential above –50 mV. By contrast, at more negative potentials, IPSC peak amplitudes deviated increasingly from the linear behavior (Fig. 4b), thereby clearly showing pronounced outward rectification. This was even stronger during the slow decay phase of the IPSCs measured at 30 ms after stimulus onset.

Light-activated IPSCs from SOM- and NOS-positive interneurons showed a very similar voltage-dependent conductance (Fig. 4c–f). In contrast to dendritic IPSCs, fast perisomatic IPSCs evoked in PV-ChR2 mice showed a more linear behavior with substantially less rectification (Fig. 4g, h). The rectification index (RI) was quantified as the ratio of the conductance obtained from the linear fit to the outward currents above –40 mV divided by the value measured at –82 mV close to the resting potential (1 = no deviation). Outward rectification was significantly larger in GABAergic synapses formed by NOS- (RI = 2.7 ± 0.2, *n* = 9, *P* < 0.001) and SOM interneurons (RI = 2.8 ± 0.2, *n* = 9, *P* = 0.001) than in perisomatic synapses formed by PV interneurons (1.8 ± 0.1, *n* = 8, Fig. 4i). Rectification was specific to GABAergic synapses, as there was no voltage-dependent rectification of AMPAR-mediated EPSCs evoked in distal SR (Supplementary Figure 5).

Plotting the GABAergic conductance of dendritic IPSCs at the peak and at 30 ms against voltage revealed a steep transition around –50 mV from a low conductance to a high conductance state. The outward rectification at 30 ms was more pronounced than at the peak, generating a 4-fold larger conductance at positive potentials than at rest (Fig. 4j). This indicates that not only the peak amplitude, but also gating kinetics might be voltage dependent (see below). To assess the voltage-dependent rectification of α5-GABA_A_Rs, we applied L-655,708 in slices from NOS-ChR2 animals and subtracted the drug-resistant light-evoked currents from the currents in ACSF (α5-GABA_A_R-IPSC, Fig. 4k). For better long-term stability of low Rs recordings (Rs < 12 MΩ), these experiments were performed at room temperature, generating similar rectification of IPSCs, as tested with SR stimulation similar to experiments in Fig. 4b (RI = 2.89, *n* = 10 at 22 °C). As shown in Fig. 4k, the outward rectification of α5-GABA_A_R-IPSC relative to –80 mV is even stronger (RI = 4.1) than what was obtained for NOS interneurons in control (RI = 2.7). This observation is consistent with two different populations of synaptic GABA_A_Rs, as suggested by previous pharmacological experiments (Fig. 1e, f), consisting of faster more linear non-α5-GABA_A_Rs and slower highly nonlinear outward-rectifying receptors formed by α5-subunit-containing GABA_A_Rs.

Taken together, these data suggest that NOS and SOM interneurons recruit outward-rectifying α5-GABA_A_Rs, which generate only about 25% of their maximal conductance at rest, corresponding to a nonlinear rectification index of 4. Assuming that the fast α5-NAM-insensitive receptors show no rectification at all, this would indicate that the maximal peak conductance is 0.5*1 + 0.5*4 = 2.5-times larger than at rest, similar to the actually measured peak rectification in SOM and NOS cells without blockers (Fig. 4i). As a result, activated α5-GABA_A_Rs in SOM and NOS interneuron–pyramidal cell synapses contribute about 80% ( = 2/2.5) to the total peak conductance at depolarized potentials.

### SOM- and NOS-interneuron-induced IPSCs show slow voltage-dependent kinetics

**Fig. 5:**
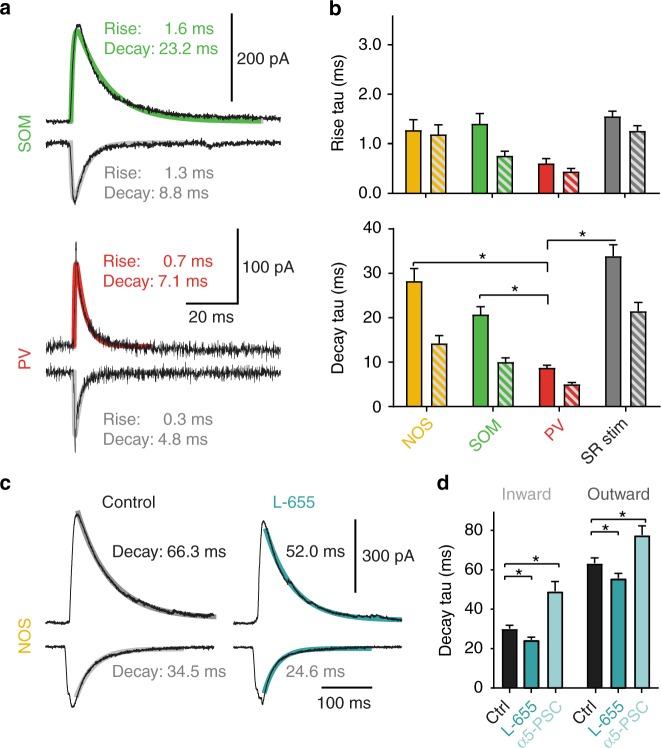
Slow PSC kinetics of dendritic GABAergic inputs. **a** Representative mean IPSCs generated by optogenetically activated inputs from SOM (top) and PV interneurons (bottom). The internal Cs-gluconate-based solution had a realistically low chloride concentration of 8 mM. Outward currents were measured at –10 mV, inward currents at –82 mV. Biexponential fits to rising and falling phases of IPSCs are indicated. **b** Group means of rise and decay *τ*. Outward currents are indicated by solid colors, inward currents by striped bars. Note the slower rise and decay *τ* for all dendritric inputs compared to PV-ChR2-evoked perisynaptic IPSCs. The significant differences in outward decay *τ* are indicated (all: *P* < 0.001; two- sample *t* test, NOS: *n* = 9; SOM: *n* = 9; PV: *n* = 8; SR stim: *n* = 11). **c** Representative mean IPSCs optogentically activated from NOS interneurons before and after application of the α5-IA L-655,708 (L-655, 50 nM). Outward currents were measured at –10 mV, inward currents at –90 mV. Monoexponential fits to the decay phase of IPSCs are indicated. Note the faster decays after application of L-655,708. **d** Group means of decay *τ* for NOS-ChR2- evoked IPSC before and after L-655,708 and the α5-GABAR-mediated difference current. Decay *τ* significantly sped up after application of L-655,708 for both inward (*P* < 0.001; paired *t* test, *n* = 10) and outward currents (*P* < 0.001). Control decay *τ* was also significantly faster than for the α5-GABAR-mediated difference current (both: *P* < 0.05, *n* = 7). Recording temperature *T* ≈ 33 °C for **a**, **b** and *T* ≈ 22 °C for **c**, **d**

The experiments above indicated that in addition to the peak conductance, also the time course of IPSCs might be voltage dependent. Therefore, we have analyzed rise and decay time course of different interneuron-evoked IPSCs at −82 and −10 mV (Fig. 5a, b). In general, the decay of the more relevant GABAergic outward currents was slower than inward currents for all three types of interneurons. Furthermore, dendrite-targeting interneurons showed slower IPSC decay *τ* for outward (NOS: 28.3 ± 2.8 ms, *n* = 9, SOM: 20.8 ± 1.7, *n* = 9) as well as inward currents (NOS: 14.2 ± 1.8 ms, SOM: 10.0 ± 0.9 ms) relative to the faster PV-IN-evoked responses in outward (8.7 ± 0.6 ms, *n* = 8, both: *P* < 0.001, two-sample *t* test) and inward direction (5.0 ± 0.4 ms, *n* = 8, both: *P* < 0.001), respectively. The slow decay of dendritic PSCs was specific to GABAergic synapses, as the decay of AMPAR-mediated inward PSCs evoked close to SP or SLM were 5.4 ± 0.6 ms (*n* = 7) and 6.2 ± 0.5 ms (*n* = 7), respectively, under the same recording conditions.

To test whether the α5-GABA_A_Rs contribute to the slow kinetics of dendritic GABAergic synapses, NOS-evoked responses were measured before and after application of L-655,708 (Fig. 5c, d). For better long-term stability of low Rs recordings, these experiments were performed at room temperature in contrast to experiments in Fig. 5a, b. Both inward and outward currents were significantly faster after drug application (*P* < 0.001, *n* = 10). Furthermore, the drug-sensitive α5-IPSC component was 2.0 ± 0.2-fold and 1.4 ± 0.2-fold (*n* = 7) slower than the drug-insensitive current for inward and outward currents, respectively.

Taken together, these data suggest that α5-GABA_A_Rs show slower gating kinetics than α5-NAM-insensitive receptors and contribute to the slow time course of SOM- and NOS-interneuron-evoked IPSCs.

### Synaptic α5-GABA_A_Rs effectively inhibit NMDAR activation by matched time course and voltage dependence

**Fig. 6:**
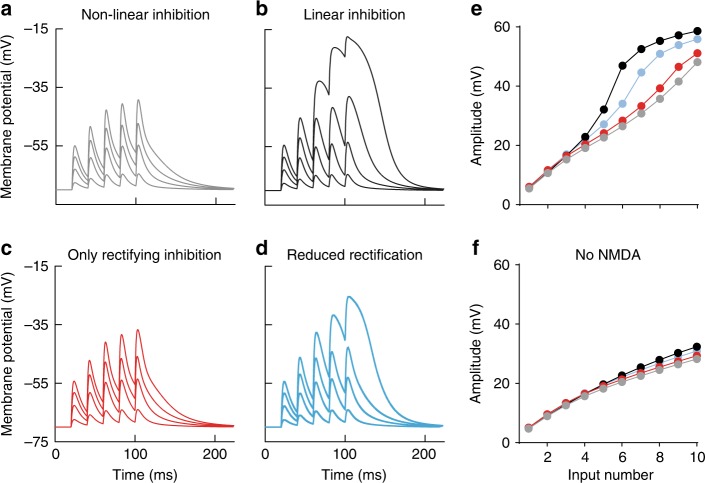
Nonlinear outward rectification of GABARs effectively counterbalances NMDAR activation in a dendritic branch model. **a** Family of voltage traces of burst activation of an increasing number of glutamatergic inputs (1, 3, 5, and 7; 0.14 nS each) in the presence of one GABAergic SOM-like model synapse that exhibits nonlinear outward rectification as observed experimentally (Supplementary Fig. 4; 0.28 nS at rest, 0.7 nS max). **b** Responses to the same stimulation when the GABAR conductance did not show a voltage-dependent increase and was constant (0.28 nS). **c** Responses when only the outward-rectifying conductance of the GABAR was active (0.14 nS at rest, 0.56 nS max). **d** Responses when the outward-rectifying conductance of the GABARs was reduced by 50% (0.21 nS at rest, 0.42 nS max). **e** The dependence of the peak burst amplitude on glutamatergic input number. Note the near-linear increase of the burst amplitude for nonlinear inhibition and the strongly nonlinear behavior for reduced or removed outward rectification of inhibition. **f** Dependence of the peak burst amplitude on input number when only AMPARs were present in the glutamate synapses

Why are dendritic α5-GABA_A_Rs so effective regulators of NMDAR activation and dendritic depolarization? To address this question, we developed computational models of synaptic GABA_A_ and glutamate receptors, including α5-GABA_A_Rs and NMDARs constrained by experimental recordings using the NEURON simulation program (Supplementary Figure 6 and Supplementary Figure 7). As a first step, we modeled a single dendritic branch with 100-µm length and 2-µm diameter. In the center of this dendrite, 1–10 glutamatergic synapses were implemented containing AMPA and NMDARs similar to previously published models^35^. Furthermore, a dendritic GABAergic synapse was generated which recruits 80% nonlinear outward-rectifying α5-GABA_A_Rs (decay 30 ms) and 20% linear GABA_A_Rs (decay 15 ms, see Methods), generating a conductance ratio of 50%/50% at resting membrane potential. Similar to experimentally determined properties of SOM- and NOS-interneuron-induced inhibition, this generates a voltage-dependent decay and a peak rectification of 2.5 at depolarizing potentials (0.5*1 + 0.5*4 = 2.5, Supplementary Figure 7). The unitary GABAergic peak conductance was adjusted according to SOM-interneuron-mediated uIPSCs reported by Maccaferri et al.^17^, which matched a typical excitation–inhibition balance of 1:1 in our dendrite when five glutamatergic synapses are activated. The synapses were stimulated in brief bursts similar to our experimental paradigm (5@50 Hz, see Methods) with an increasing number of active synapses in different bursts (Fig. 6).

As a consequence of increasing synapse number, the peak amplitude of burst PSPs increased linearly with co-activation of our modeled dendritic GABAergic synapse containing α5-GABA_A_Rs (Fig. 6a, e, gray). When the rectification of the α5-component was disabled, dendritic depolarization was strongly supra-linear (Fig. 6b, black). By contrast, silencing the non-rectifying component was much less effective (Fig. 6c). Furthermore, when the rectifying component was reduced by 50%, simulating the application of the α5-NAM, depolarization changed already to a strongly nonlinear regime (Fig. 6d, e, blue). The nonlinear dendritic depolarization of burst PSPs in the absence of rectifying α5-GABA_A_Rs was fully dependent on NMDARs, as it was absent with AMPAR-only synapses (Fig. 6f). This shows that the outward rectification of α5-GABA_A_Rs provides inhibitory GABAergic conductance on demand, perfectly suited to counterbalance voltage-dependent NMDAR activation with minimally affecting small AMPAR-mediated PSPs.

**Fig. 7:**
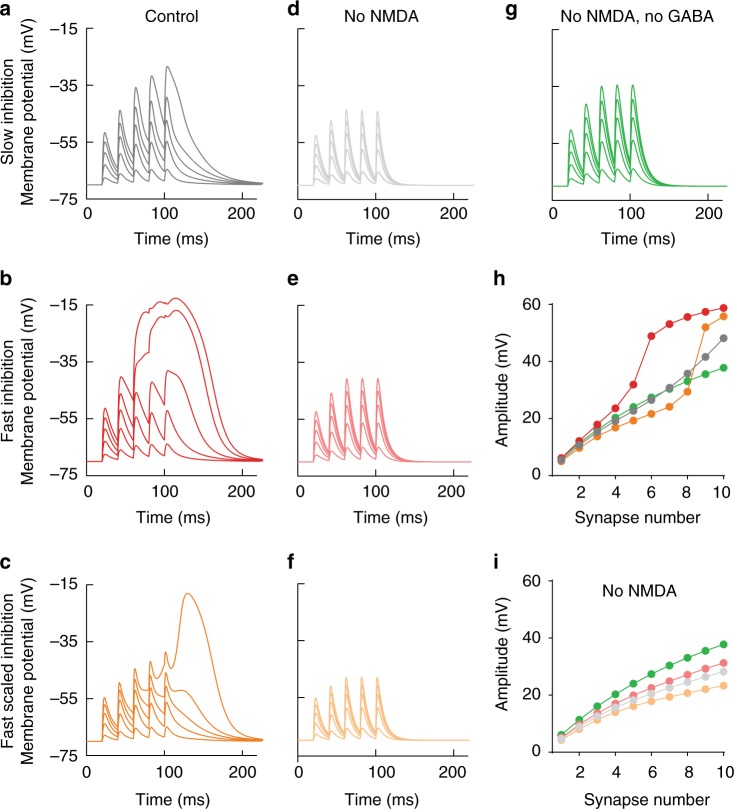
Slow kinetics of outward-rectifying GABARs allow for effective inhibition of NMDAR-mediated electrogenesis. **a** Family of voltage traces of burst activation of an increasing number of glutamatergic inputs (1, 3, 5, 7, and 9; 0.14 nS each) in the presence of one nonlinear SOM-like GABAergic model synapse with slow kinetics. **b** Responses to the same stimulation when the nonlinear GABAergic model synapse has five-times faster kinetics (rise *τ* = 0.2 ms; decay *τ* = 6 ms) similar to PV-basket-cell-mediated perisomatic inhibition. **c** The amplitude of the conductance was additionally scaled by a factor of 5 (from 0.7 to 3.5 nS) to compensate for the decreased inhibitory charge when kinetics are faster. Note that despite the same total integral conductance, fast scaled inhibition was less effective at preventing NMDAR-mediated dendritic spikes. **d**–**f** The same simulations when only AMPARs were present in glutamate synapses. Note the larger effect of fast scaled inhibition onto AMPAR-mediated depolarization. **g** Pure AMPAR responses without any inhibition. **h** The dependence of the peak burst amplitude on glutamatergic input number. Note the strongly nonlinear behavior for both forms of fast inhibition. **i** Dependence of the peak burst amplitude on input number when only AMPARs were present in the glutamate synapses

To investigate the impact of α5-GABAR gating kinetics on dendritic integration, we scaled the gating of α5-GABA_A_Rs by a factor of 5 (decay *τ* = 6 ms), resembling the kinetics of PV-interneuron synapses (Fig. 7). Faster GABAR gating kinetics generated strongly nonlinear NMDAR activation (Fig. 7b). As the faster decay time course also reduced the total inhibitory charge, we compensated for this effect by a 5-fold increase in peak conductance amplitude (Fig. 7c). This dramatic increase in the peak amplitude could not prevent nonlinear NMDAR activation because the fast GABA_A_Rs closed much earlier than NMDARs (decay *τ* = 35 ms). As a consequence, they escape the GABAergic inhibition and generate a dendritic NMDA spike. While the slow α5 gating kinetics only slightly affect AMPAR-mediated burst PSPs, the effect on AMPAR PSPs was larger with faster kinetics (Fig. 7d–g–i). Taken together, the slow and outward-rectifying dendritic GABA_A_Rs are necessary to match the slow time course and nonlinear voltage dependence of NMDARs.

To simulate a more complex scenario, we used a detailed compartmental cable model of a CA1 pyramidal cell, including full-scale excitatory and inhibitory synapses derived from large-volume array tomography^35^. In this model, simulating many active glutamatergic synapses during synchronous perforant-path and Schaffer-collateral stimulation reproduced our experimental findings from Fig. 2 assuming that all dendrite-targeting synapses in SR had similar properties to the experimentally determined NOS- and SOM-interneuron synapses (Supplementary Figure 8). The increase in somatic burst PSP by silencing 50% of the α5-GABA_A_Rs was similar to our experimental results obtained after α5-NAM application, explaining the strong effect on the NDMAR-dependent depolarization (Supplementary Figure 8C, soma with 125 inputs).

As some α5-GABAR are localized extrasynaptically, an α5-NAM-mediated change in tonic inhibition (~0.5 nS, Supplementary Figure 2) could potentially contribute to an increase in burst PSP amplitude. However, modeling a 50% decrease of tonic inhibition to simulate the effect of α5-NAM application without changing phasic inhibition, increased PSP amplitude by only 2% (Supplementary Figure 9B, blue vs. black). To obtain an increase of about 30% similar to our burst PSP experiments in Fig. 2, a tonic conductance of 24.8 nS would be necessary, corresponding to 100-times larger conductance density than that estimated from our measurements. This indicates that modulation of synaptic α5-GABA_A_Rs was necessary and sufficient to explain our measured effects of α5-NAM on dendritic PSP integration, while modulation of tonic inhibition played a minor role.

**Fig. 8:**
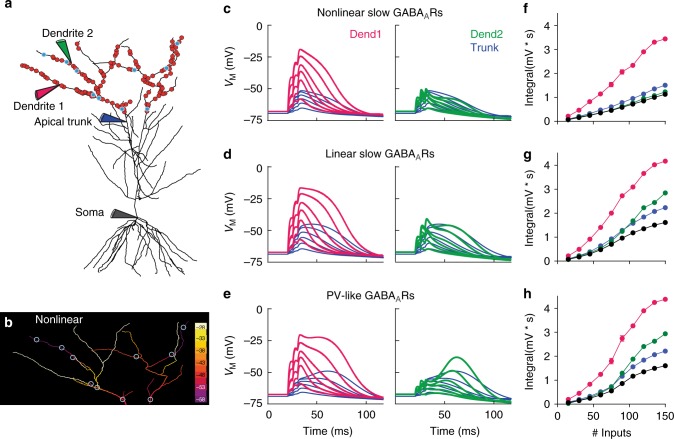
SOM-interneuron-evoked nonlinear GABAergic inhibition powerfully regulates NMDAR-mediated dendritic spikes. **a** Computational pyramidal-cell model with stimulated glutamatergic (red) and GABAergic synapses (blue). **b** The local membrane potential as a result of glutamatergic and GABAergic (open circles) inputs at 20 ms after a short burst stimulation (3@200 Hz) is shown as a heat map across the tuft dendrites. **c** Membrane potential responses from the same simulation are shown for the left apical dendritic trunk (blue traces) and two tuft dendrites, one without inhibition (left, magenta traces) and one with strong inhibition (right, green traces). Recording sites of the local membrane potential are indicated by colored pipettes in **a**. The family of different voltage traces show brief-burst activation of an increasing number of glutamatergic inputs. The number of active synapses during the first stimulus (15, 45, 75, 105, 135, and 165) represent about 1–10% of all available tuft synapses. **d** When the GABAR conductance is constant, membrane potential responses exhibit larger NMDAR-mediated plateau depolarizations in both the disinhibited and the inhibited tuft dendrite. **e** Inhibition with PV-like dynamics using 5-fold faster time course of the slow component (rise *τ* = 0.2 ms; decay *τ* = 6 ms) and 5-times larger peak conductance, fails to prevent an NMDAR-mediated dendritic spike in the inhibited tuft dendrite if the number of glutamatergic inputs exceeds 5.1% active synapses (>75 of 1464 distributed over the whole tuft). **f**–**h** The dependence of the burst PSP integral measured in the different compartments on glutamatergic input number for all three conditions. Each point is the mean of five simulations with randomized glutamatergic synapse locations, while the location of GABA synapses was kept constant. The SEM is indicated. magenta: dend1; green: dend2; blue: apical trunk; black: soma

Next, we investigated specifically the interaction of glutamatergic entorhinal inputs in SLM with the activity of SOM- and NOS-interneuron-induced synaptic inhibition during high-frequency synaptic activation. This is a condition that is thought to be crucial for the induction of local NMDAR-mediated spikes, but that is not easily accessible to physiological experimental approaches. Therefore, we simulated brief-burst activity (3@200 Hz) in a randomly selected subset (1–10%) of the perforant-path synapses with simultaneous activation of 3.5% randomly selected GABAergic synapses in SLM (Fig. 8a). We separately analyzed burst-evoked potentials in different branches of the dendritic tree with and without active GABAergic synapses using different functional properties of GABA_A_Rs (Fig. 8b–e). Simulating the physiologically determined slow nonlinear outward-rectifying GABA_A_Rs efficiently decreased burst potentials in fine branches with active GABAergic synapses (dendrite 2) versus non-inhibited branches (dendrite 1, Fig. 8c, f, green vs. magenta). By contrast, when all GABA_A_Rs were linear, depolarization in branches was much less controlled by the inhibitory synapses generating large dendritic potentials (Fig. 8d, g). Finally, when GABAR gating kinetics were changed to PV-like conditions with 5-fold faster decay and 5-times larger peak amplitude, NMDARs escaped from inhibition and generated dendritic NMDA spikes after GABARs had closed (Fig. 8e, h). Apparently, removing outward rectification and scaling kinetics to PV-like values not only reduced the ability to inhibit dendritic NMDA spikes. It also rendered voltage signals in different branches (magenta vs. green) more similar to each other. This indicates that the functional properties of α5-GABA_A_Rs allow for local signal processing, specifically inhibiting NMDAR recruitment in distinct fine dendritic branches. Therefore, slow and outward-rectifying α5-GABA_A_Rs can powerfully prevent nonlinear NMDAR activation in a spatiotemporally controlled manner in fine dendrites of CA1 pyramidal cells.

Taken together, the computational analysis shows that the experimentally determined effects of the α5-NAM on NMDA-dependent dendritic depolarizations cannot be reproduced and cannot be predicted by classical linear GABA_A_-receptor function. Instead, it shows that the outward rectification of synaptic α5-GABA_A_Rs is essential to counteract the slow and voltage-dependent activation of NMDARs above –50 mV. As α5-GABA_A_R conductances are 4-fold smaller at resting membrane potential, the synapses generate minimal inhibition with small PSPs but provide large shunting conductances on demand, during stronger depolarization generated by brief-burst activity. This keeps shunting local and energy efficient during small EPSPs. Most importantly, it allows dendrite-targeting interneurons to powerfully control NMDAR-dependent burst firing and synaptic plasticity.

## Discussion

Taking advantage of targeted optogenetic stimulation of different CA1 interneuron subtypes in combination with the highly selective α5-NAM RO4938581, we found that SOM and NOS interneurons targeting distal CA1-pyramidal-cell dendrites in SLM preferentially activate α5-GABA_A_Rs which contribute 50–80% to the peak conductance. The IPSCs mediated by α5-GABA_A_Rs showed a slow time course (decay ≈30 ms) and a nonlinear outward-rectifying voltage dependence (*V*_50_ ≈ –50 mV), which matches the properties of synaptic NMDARs in CA1 pyramidal cells. By contrast, perisomatic inhibitory inputs from PV interneurons did not only show faster kinetics, but also substantially less rectification and a much more linear behavior. We have found that synaptic α5-GABA_A_Rs preferentially control NMDA-receptor-mediated synaptic depolarization, while non-α5-receptors affect NMDA- and non-NMDA-PSPs to a similar extent. These findings could be reproduced by computational modeling of synaptic integration in pyramidal-cell dendrites, which further showed that the properties of synaptic α5-GABA_A_Rs are ideally suited to temporally and spatially control the nonlinear NMDAR activation in fine dendritic tuft branches. By contrast, PV-basket-cell-like synapses were strikingly less effective in controlling NMDAR activation when placed in distal dendrites. Most importantly, the inhibition of dendritic NMDA spikes via α5-GABA_A_R-containing synapses provides a mechanistic basis for the powerful control of NMDAR-dependent burst firing and synaptic plasticity in CA1 pyramidal cells by dendrite-targeting interneurons.

The involvement of α5-GABA_A_Rs in phasic synaptic transmission has been a reoccurring theme for many years^18–20,39^. Furthermore, preferential synaptic localization of these receptors has been documented by electron microscopy^23^ and substantial co-immunoprecipitation with gephyrin^40^. Nevertheless, the involvement of α5-GABA_A_Rs in phasic synaptic transmission has remained controversial, in contrast to the well-accepted role of α5-GABA_A_Rs in tonic inhibition^22,24,31^. The reasons for this may be manifold. First, spontaneous and miniature IPSCs are heavily biased toward contribution of fast perisomatic GABAergic synapses with negligible α5-subunit contribution. Small and slow spontaneous IPSCs originating from distal dendritic synapses are much more difficult to detect in the background noise. Second, to activate tonic currents, typically GABA-uptake is blocked or brain slices are perfused with low concentrations of 1–5 µM GABA. This will not only activate extrasynaptic α5-GABA_A_Rs, but will also activate the sub- and perisynaptic high-affinity GABA_A_Rs and partially inactivate and desensitize them, thereby decreasing the contribution of α5-GABA_A_Rs to phasic inhibition. Finally, although there is good evidence for some contribution of α5-GABA_A_Rs to unitary IPSCs obtained by paired recordings in the neocortex^39^, precise quantification has been hampered by the limited selectivity and availability of GABA_A_R subtype-selective blockers^41^. Nevertheless, using an inverse agonist preferentially acting on α5-GABA_A_Rs donated by Merck, Ali and Thomson^39^ found a 35% reduction of dendritic IPSPs in cortical pyramidal cells (recorded at −55 mV), evoked by putative Martinotti cells. By contrast, multipolar basket cells targeting soma and proximal dendrites were reported to be insensitive to the α5-selective inverse agonist. Therefore, these data would be fully consistent with the notion that dendrite-targeting SOM interneurons in the neocortex also recruit α5-GABA_A_Rs very similar to hippocampal SOM interneurons.

Our experiments clearly show a preferential contribution (50–80%) of α5-subunit-containing GABA_A_Rs to the peak amplitude of PSCs in synapses from SOM and NOS interneurons onto CA1 pyramidal cells. However, the precise contribution of synaptic versus perisynaptic GABA_A_Rs is not fully clear at present. Evidence from electron microscopy suggests that α5-GABA_A_Rs are enriched in synaptic membranes of CA1 pyramidal cells and show an 8-fold lower density in perisynaptic and a 20-fold lower density in the extrasynaptic cell membrane^23^, indicating that the contribution of synaptic receptors to measured PSCs is likely to be dominant. Future experiments will have to show the exact contribution of sub-synaptic and perisynaptic α5-GABA_A_Rs to synaptic signaling of SOM and NOS interneurons under different physiological conditions.

Synaptic NMDARs are believed to be the key regulators for cooperative synaptic integration, generation of dendritic spikes, and synaptic plasticity^42,43^. They generate local dendritic NMDA spikes dependent on both, postsynaptic voltage within individual dendritic branches and presynaptic glutamate release at different individual synapses, which allows for local and complex signal processing within the dendritic tree, leading to input-specific synaptic plasticity^10,43,44^.

SOM interneurons have been shown to control dendritic NMDAR activation and NMDAR-dependent burst firing much more effectively than PV-neurons^1,6,7^. By contrast, PV-neurons control precise timing of AP output. These distinct functional properties may be in part due to the highly dynamic excitatory recruitment of SOM interneurons during pyramidal-cell activity^45,46^ and a specifically structured spatial organization of inhibitory GABAergic synapses^35^. However, our results provide evidence for an additional functional specialization of SOM- and NOS-interneuron synapses with slow kinetics and a voltage-dependent conductance profile that matches the properties of NMDARs. The nonlinear outward rectification provides a powerful inhibitory conductance on demand, dependent on local dendritic activity levels. This is not only energy efficient, as it minimizes current flow when it is not required. It also provides a powerful control over the local voltage in fine dendritic branches at times of high activity (Fig. 8) and thereby precise control over localized nonlinear electrogenesis and finally AP output. This novel mechanism may underlie the reported control of dendritic spikes in individual dendritic branches^4^ and sharpening of stimulus selectivity of pyramidal cells by SOM interneurons^2^. In turn, synaptic plasticity can be effectively gated by disinhibition via feedforward VIP-cell-mediated inhibition onto SOM interneurons^3,47^.

Our new findings also reinforce the concept that α5-GABA_A_Rs represent promising drug targets for the treatment of several neurological and psychiatric conditions^24,41,48^. A number of compounds that selectively target α5-GABA_A_Rs, as either negative or positive modulators have been described^49^. It has already been shown that α5-NAMs may be beneficial in states of hyperinihibition in animal models of Down syndrome^29^. Our data suggest that facilitation of distal dendritic integration and generation of dendritic NMDA spikes probably contribute to these functional improvements. The same mechanisms may also underlie the powerful anti-depressive effect of α5-NAMs recently described in rodents^50,51^. On the other hand, α5-PAMs might help to control enhanced E/I balance and associated unspecific synaptic plasticity. For example, it was suggested that autism spectrum disorder (ASD) is associated with enhanced glutamate transmission, reduced GABA transmission, and disturbed cortical connectivity, which might be partially mediated via reduced α5-GABA_A_R activity^52,53^. Interestingly, α5 knockout mice have been reported to exhibit ASD-like behavioral phenotypes, consistent with a major role of α5-GABA_A_Rs in the control of input-specific NMDAR-dependent synaptic plasticity^54,55^.

Taken together, our results show that dendrite-targeting SOM and NOS interneurons preferentially activate nonlinear-rectifying α5-GABA_A_Rs with slow kinetics, which match the functional NMDAR properties and thereby mediate powerful control of NMDAR activation, nonlinear dendritic integration, and AP firing. This novel mechanism not only explains the powerful impact of dendrite-targeting interneurons on plasticity and cognition, but also opens new avenues for development of new drug treatments of cognitive disorders.

## Methods

### Animals

For this study, wild-type mice (C57BL/6) and the following transgenic mouse lines were used: PV-Cre (B6;129P2-Pvalbtm1(cre)Arbr/J), SOM-Cre (SST tm2.1(cre)Zjh/J), NOS-Cre (B6;129S-Nos1tm1.1(cre/ERT2)Zjh/J), LoxP-ChR2 (B6.Cg-Gt(ROSA)26Sortm32(CAG-COP4*H134R/EYFP)Hze/J), and VGAT-ChR2-YFP mice (B6.Cg-Tg(Slc32a1-COP4*H134R/EYFP)8Gfng/J). All transgenic mice were obtained from The Jackson Laboratory and were bred by pairing hemizygous transgenic mice with wild-type C57BL/6 animals. For optogenetic experiments, offspring from crosses of homozyguous Cre mice with homozyguous LoxP-ChR2 mice and hemizyguous VGAT-ChR2-YFP mice were used.

Mice were housed in groups of up to five animals in standard individually ventilated cages in standard laboratory conditions with a 12-h light/dark cycle, and access to food and water ad libitum. All experiments were approved by the Basel Cantonal Committee on Animal Experimentation according to federal and cantonal regulations.

### Slice preparation for patch-clamp recordings

Adult 5- to 10-week-old male and female mice were anesthetized with isoflurane (4% in O_2_, Vapor, Draeger) and killed by decapitation, in accordance with national and institutional guidelines. In order to increase cell viability for single-cell patch-clamp recordings, animals were exposed to oxygen-enriched atmosphere for 10 min prior to decapitation. Slices were cut as previously described^56,57^. Briefly, the brain was dissected in ice-cold sucrose-based solution at about 4 °C. Horizontal 350-μm-thick hippocampal brain slices were cut at an angle of 20° to the dorsal surface of the brain along the dorsoventral axes of the hippocampus using a Leica VT1200 vibratome. For cutting and storage, a sucrose-based solution was used, containing 87 NaCl, 25 NaHCO_3_, 2.5 KCl, 1.25 NaH_2_PO_4_, 75 sucrose, 0.5 CaCl_2_, 7 MgCl_2_, and 10 glucose (equilibrated with 95% O_2_/ 5% CO_2_). Slices were kept at 35 °C for 30 min after slicing and subsequently stored at room temperature until experiments were performed.

### Patch-clamp recordings

CA1 pyramidal neurons were visually identified in the pyramidal-cell layer close to the border of SR using infrared differential interference contrast (IR‐DIC) video microscopy. Slices were continuously superfused with artificial cerebrospinal fluid (ACSF) at near-physiological temperature (32–33 °C) in most experiments. Experiments with optogenetic stimulation for Fig. 3, Figs. 4k and 5c, as well as supplementary experiments in Supplementary Figure 1 and Supplementary Figure 2 were performed at 20–22 °C. The ACSF contained (in mM) 125 NaCl, 25 NaHCO_3_, 25 glucose, 2.5 KCl, 1.25 NaH_2_PO_4_, 2 CaCl_2_, and 1 MgCl_2_ (equilibrated with 95% O_2_/5% CO_2_). Patch pipettes were pulled from borosilicate glass tubing with a 2.0-mm outer diameter and 0.5-mm wall thickness (Hilgenberg GmbH, Malsfeld, Germany) on a Flaming-Brown P-97 puller (Sutter Instruments, Novato, USA).

For current-clamp recordings, patch pipettes (4–7 MΩ) were filled with a solution containing (in mM) 135 KMeSO_4_, 4 KCl, 10 EGTA, 10 Hepes, 2 MgCl_2_, 2 Na_2_ATP, 0.3 GTP, and 0.2% biocytin adjusted to pH 7.3 with KOH.

For voltage-clamp recordings of large inward IPSCs (Figs. 1, 3, and Supplementary Figure 2), low-resistance patch pipettes (2–4 MΩ) were filled with a CsCl-based solution containing (in mM) 100 CsCl, 40 Cs-gluconate, 10 EGTA, 10 Hepes, 2 MgCl_2_, 2 Na_2_ATP, 0.3 GTP, and 5 QX314 adjusted to pH 7.3 with CsOH. For all other voltage-clamp recordings (Figs. 4, 5, Supplementary Figures 1, 5, and 6), patch pipettes (2–4 MΩ) were filled with a Cs-gluconate-based solution with low chloride (8 mM) containing (in mM) 135 CsGluc, 2 CsCl, 10 EGTA, 10 Hepes, 2 MgCl_2_, 2 Na_2_ATP, 2 TEA-Cl, and 5 QX314 adjusted to pH 7.3 with CsOH.

Voltage and current signals were measured with a Multiclamp 700 A amplifier (Molecular Devices, Palo Alto, CA, USA), low-pass filtered with a cutoff frequency of 8 kHz, and digitized at 20 kHz using a CED Power 1401 interface (Cambridge Electronic Design, Cambridge, UK). Bridge balance was used to compensate the series resistance (*R*_S_ = 10–40 MΩ) in current-clamp recordings. Series resistance (<20 MΩ) in voltage-clamp experiments was monitored online and experiments were discarded if *R*_S_ changed more than 20%. In voltage-clamp recordings to examine voltage dependence and time course of postsynaptic currents (Figs. 4, 5, Supplementary Figures 5, 6), whole-cell series-resistance (5–12 MΩ) compensation was used (60–80% correction and prediction). In these experiments, PSCs were recorded at increasing membrane potentials with voltage commands ranging from −94 to +26 mV in 12-mV steps or –90 to + 10 mV in 20-mV steps. Data acquisition was controlled using IGOR Pro 6.31 (WaveMetrics, Lake Oswego, Oregon) and the CFS library support from CED (Cambridge Electronic Design, Cambridge, UK).

### Tonic inhibition and spontaneous IPSCs

In CA1 pyramidal cells, a picrotoxin (PTX)-sensitive tonic current contributed 9.6 ± 4.2 pA (*n* = 3) to the holding current during voltage-clamp recordings with CsCl-based internal solution. In order to study the contribution of α5-GABA_A_Rs to tonic currents by pharmacological means, larger GABA-dependent tonic currents were evoked by bath-application of 5 µM GABA in the presence of 25 µM AP5 and 10 µM CNQX similar to previously published studies^58^. In some experiments, 200 nM gabazine was used to separate tonic current from fast spontaneous IPSCs (Supplementary Figure 2G).

### Assessment of cellular excitability

To evaluate the cellular properties, 1-s-long current pulses of increasing amplitude (steps of 25 pA) were injected during current-clamp recordings. To assess changes in the input resistance, 100-ms-long current pulses were applied every 5 s. The voltage responses were averaged over 3 min before the start and over 3 min after the completion of a 5-min wash-in phase. To assess changes in the excitability, 30-ms-long current pulses of 12 different amplitudes (step size of 5 pA) around the AP current threshold were repeatedly applied (6–10 repetitions, interstimulus interval of 3 s) before and more than 5 min after the wash-in of the α5-NAM (1 µM). In all current-clamp protocols, the resting membrane potential was kept constant close to the initial potential of about −70 mV by small constant current injections throughout the experiment.

### Extracellular synaptic stimulation

For stimulation of synaptic inputs, 4–6 MΩ pipettes filled with HEPES-buffered Na^2+^-rich solution were used to apply brief negative current pulses (5–40 µA, 200 µs). In order to stimulate local fibers, the pipettes were placed close to the recorded cell at a distance of <200 µm in either SR, SLM, or SP. For voltage-clamp recordings, the SP stimulation strength was generally reduced to ≤10 µA to ensure recruitment of local perisomatic/basket-cell axons. In voltage-clamp recordings of IPSCs with a CsCl-based internal solution (Figs. 1, 3), the neuron was clamped at −70 mV, and the GABAR-mediated inward currents were measured in the presence of 10 µM NBQX and 25 µM AP5. Stimulus artifacts have been truncated in some figures for clarity.

### Channelrhodopsin-assisted localization of α5-GABA_A_Rs

A diode laser (DL-473, Rapp Optoelectronic) was coupled to the epifluorescent port of the microscope (Zeiss Examiner, equipped with a 63x NA1.0 water immersion objective; Carl Zeiss Microscopy GmbH, Jena, Germany) via fiber optics. The laser was controlled via TTL pulses. For the optogenetic activation of the axon of specific interneuron subpopulations, the field of view was shifted, such that the recorded neuron and GABAergic boutons were outside the illuminated area, using laser intensities of 0.1–0.5 mW for 5 ms.

For sCRACM, the field of illumination was restricted to an area with a diameter of 20–50 μm located in the center of the field of view. Five light flashes (1 ms, 473 nm) were applied at 500 Hz to evoke a PSC. Stimulation was targeted to GABAergic boutons at the soma or to the apical dendrite 200–300 μm from the cell body. The dendrite was visualized by epifluorescence of Alexa-594 (10 μM) in the internal solution. Stimulation intensities varied from 0.1 to 0.5 mW for somatic stimulations, and 0.5–3 mW for dendritic stimulations. To ensure localized activation of presynaptic terminals without axonal AP propagation, experiments were performed in the presence of 1 µM TTX, 75 µM 4-AP, and 1 µM CGP. To increase the stability of evoked responses, sCRACM was performed at room temperature (20–22 °C) and the ACSF contained (in mM) 1.5 CaCl_2_ and 1.5 MgCl_2_. Stimulations were repeated at an interval of ≥ 55 s to minimize rundown of recorded responses. Only experiments with a minimum of five stable responses before and after the wash-in of α5-NAM (1 µM) were included in the analysis.

### Drugs and reagents

4-Aminopyridin (4-AP, 100 mM; Merck), D-AP5 (50 mM; Tocris), and tetrodotoxin (TTX, 1 mM; Alomone labs) were dissolved in water. Picrotoxin was dissolved at 50 mM in ethanol. CGP 54626 hydrochloride (10 mM; Tocris), CNQX (20 mM; Tocris), NBQX (20 mM; Tocris), L-655,708 (1 mM; Tocris), and the α5-NAM RO4938581 (10 mM; F. Hoffmann-La Roche) were dissolved in DMSO.

### Data analysis and statistics

Analysis of patch-clamp data was performed offline using the open-source analysis software Stimfit^59^ (https://neurodroid.github.io/stimfit) and customized scripts written in Python.

During analysis, measured amplitudes were plotted against time to check for the stability of recorded signals. Final analysis of voltage-clamp data was performed on mean waveforms averaged from 5 to 10 sweeps. Rise and decay *τ* of PSCs were obtained by fitting the sum of two exponential functions over the whole PSC waveform. For IPSCs, the decay *τ* was the weighted average of a biexponential fit only to the decay phase of the PSC starting at 95% of its amplitude. For calculations of the integral, traces were low-pass filtered (third- order Butterworth, 100-Hz cutoff) to determine the start and endpoint of the integral as the intersection of the smoothed trace with the baseline. The integral was then calculated as the sum of all values of the original trace minus the baseline in-between start and endpoint.

Voltage dependence of normalized GABAR- and NMDAR-mediated conductance (Fig. 4j, Supplementary Figures 6, 7) was calculated for each experiment individually by dividing PSC amplitudes by the difference of the voltage command from the estimated reversal potential. All conductances were normalized by the conductance measured at +14 mV for GABAR- and at +26 mV for NMDAR currents, respectively. A sigmoidal function was fitted to the scatter plot of mean normalized conductance versus voltage command. For analysis of NMDAR-mediated PSC amplitudes in Supplementary Figure 3B, the liquid junction potential was corrected by about −5 mV, leading to an average current–voltage relationship reversing at approximately at 0 mV (*E*_NMDA_ = 0.35 ± 1.12 mV, *n* = 6) as expected for a nonspecific cation conductance.

For the visualization of the outward rectification of GABAR-mediated currents, current amplitudes were normalized by the value at –70 mV (*I*_norm_). The normalized currents were fitted in GraphPad Prism6 using a sigmoidal voltage-dependent conductance:

1 $$I_{{\rm{norm}}}\left( v \right) = (v - E_{{\rm{rev}}}) \cdot (g_{{\rm{min}}} + \frac{{g_{{\rm{max}}} - g_{{\rm{min}}}}}{{1 + e^{(V_{50} - v)/{\rm{Slope}}}}})$$

with *v* representing the membrane potential, *E*_rev_ the GABA_A_ reversal potential, *g*_min_ and *g*_max_ the minimal and maximal conductance, *V*_50_ the membrane potential at half-maximal voltage-dependent conductance increase, and Slope determining the steepness of the sigmoidal, which was constrained to be at least the voltage difference between two adjacent data points.

To compare the strength of the observed rectification across different experimental conditions, we calculated a rectification index (RI) that was quantified as the ratio of the conductance obtained from the linear fit to the outward currents above −40 mV divided by the value measured at –82 mV (1 = no deviation).

To assess the impact of α5-NAM on tonic GABA currents, medians of 5-s-long epochs of the holding current were measured. The median was minimally sensitive to sporadic spontaneous IPSCs. The mean of all medians of a specific epoch was saved and compared across epochs for drug-specific effects.

The analysis of burst PSPs in current-clamp recordings was performed on single-trial data to avoid distortion by occasional AP discharge. In rare cases of AP firing, APs were digitally removed by cutting off spikes at the AP threshold, defined by the voltage slope (10 Vs^−1^) before calculating the integral.

Statistical analysis was performed in GraphPad Prism 6. Before statistical evaluation, data were always tested for normality by the Shapiro–Wilk normality test. In most instances, statistical estimations of significance of paired data, in particular normalized data relative to 100% control, were derived from paired two-tailed Student’s *t* tests. For comparisons between groups, statistical tests were two-sample two-tailed Student’s *t* tests. If standard deviations of groups appeared to be different a Welch’s correction was used. Data sets that failed the Shapiro–Wilk normality test were subsequently analyzed with the nonparametric Wilcoxon signed rank and the Mann–Whitney tests for paired and unpaired data, respectively. The significance level was set to *P*=0.05. All data are shown as mean ± s.e.m. The sample size was determined by the reproducibility of the experiments and based on our experience with similar experiments. Unless stated otherwise, the number *n* of observations indicated reflects the number of cells recorded from.

### Computational modeling

Two computational models were designed in the NEURON simulation environment^60^. The multicompartmental model and most of the mod files for various conductances were based on the model published by Bloss et al.^35^. The first model consisted of a passive piece of dendrite of 100-µm length and 2-µm diameter. We included a glutamate synapse, including an AMPAR- and a NMDAR-mediated conductance, and a GABAergic synapse at the same central location. AMPA, NMDA, and GABA conductance were modeled with exponential rise and decay using the Exp2Syn class. For the AMPA conductance, the time constant (*τ*) for rise and decay *τ* was set at 0.2 ms and 2 ms, respectively. For the NMDA conductance, the rise and decay was set to *τ*_rise_ = 3 ms and *τ*_decay_ = 35 ms, based on our experimental data for NMDA currents in the range between –60 and –20 mV (Supplementary Figure 6). The voltage-dependent magnesium (Mg^2+^) block of NMDARs was modeled as

2 $$g_{{\rm{NMDA}}}(v) = g_{{\rm{max}}}/(1 + 0.2801[{\rm{Mg}}^{2 + }] \cdot e^{ - 0.087\left( {v + 10{\rm{mV}}} \right)})$$

based on our own experimental observations (Supplementary Figure 6B inset; see the resulting current–voltage profile in Supplementary Figure 7C) and previously published single-channel recordings^61^. The extracellular magnesium concentration [Mg^2+^] was set to 1 mM according to the concentration in our ACSF.

Nonlinear GABAergic synapses that showed outward rectification as observed experimentally (Fig. 3), were modeled as the sum of two Exp2Syn class synapses in NEURON. The first component was modeled as a linear conductance and was assigned 20% of the total synaptic weight. The second rectifying conductance component was assigned 80% of the total synaptic weight. The 4-fold voltage-dependent outward rectification of this second component was modeled as

3 $$g_{\alpha 5}\left( v \right) = g_{{\rm{max}}}(0.25 + 0.75/(1 + e^{ - (v - V_{50})/V{\rm{slope}}}))$$

based on the fit to the experimentally obtained conductance profile of the slow IPSC component measured at 30 ms after the peak (Fig. 4j shows a scaled version of this curve). According to the fitted data, *V*_slope_ was set to 3 mV. The half-maximal activation was adjusted to *V*_50_ = –52 mV, which was 5 mV more negative than the fitted value (−47 mV) to correct for the liquid junction potential difference similar to the voltage dependence of NMDAR currents. Linear and outward-rectifying GABA conductance components obtained different kinetic parameters to model the voltage dependence of the decay time course of dendritic GABAergic currents (Fig. 5). For the linear GABA synapse, rise and decay *τ* were set to 0.5 and 15 ms, respectively; for the outward-rectifying GABA synapse, the rise and decay *τ* were set to 1 and 30 ms, respectively.

For the single-dendrite simulations shown in Fig. 6, a glutamate synapse (with AMPAR and NMDAR conductance) and a nonlinear GABA synapse were placed at the same central location of the dendritic branch. Maximal conductances were set to 0.14 nS for AMPAR and NMDAR conductances, and to 0.7 nS for the nonlinear GABAergic synapse to generate an E/I balance of 1:1 with 5 glutamatergic inputs. Schaffer- collateral inputs showed paired-pulse facilitation during brief-burst activity (5@50 Hz), resulting in a relative increase in EPSC amplitude during the 2nd, 3rd, 4th, and 5th pulse by a factor of 1.5 ± 0.2, 1.8 ± 0.3, 1.9 ± 0.4, and 2.0 ± 0.5 (*n* = 4), respectively. To account for this experimentally determined short-term plasticity during repetitive stimulation, the synaptic weight of glutamate synapses (both AMPAR and NMDAR conductances) was adjusted to 1.5 times the weight on the second stimulus and to 2 times the weight for all subsequent stimuli. The simulation was repeated 10 times with increasing numbers of glutamatergic inputs which were simulated by simply increasing the synaptic weight of the modeled glutamate synapse. To explore the impact of the functional properties of GABAergic synapses onto dendritic depolarization, the nonlinear model was modified in the following way: for linear inhibition (Fig. 6b), the voltage dependence of the rectifying component was turned off and its weight was fixed at 0.25 of the original maximal conductance resulting in a completely linear conductance at 40% of 0.7 nS (this reflects traditional interpretation of inward IPSCs recorded at resting potential with symmetric chloride levels); for only rectifying inhibition (Fig. 6c), the linear conductance was turned off; for simulation of the α5-NAM effect (Fig. 6d), the synaptic weight of the rectifying component was halved resulting in baseline and maximal conductances of 30% and 60% of 0.7 nS. In Fig. 6, the rise and decay *τ* of the rectifying conductance was decreased by a factor of 5 (i.e., rise *τ* = 0.2 ms, decay *τ* = 6 ms; Fig. 7b). In a second step, the synaptic weight of this component was increased by the same factor (from 0.56 to 2.8 nS) to achieve the same integral PSC (i.e., charge transfer) as with the original slow nonlinear synapse (Fig. 6c).

The second model was the multicompartmental CA1 pyramidal neuron taken from Bloss et al.^35^ and modified in the following ways. Sodium and potassium reversal potentials were set to *E*_Na_ = 55 mV and *E*_K_ = –95 mV. The specific membrane capacitance was *C*_m_ = 1 µF/cm^2^. The internal resistivity was *R*_i_ = 200 Ωcm and the specific membrane resistance was set to *R*_m_ = 60 kΩcm^2^. The delayed rectifier potassium conductance was deleted from all dendrites > 100 µm from the soma^62^. The proximal A-type potassium conductance density was set to 0.001 S/cm^2^ and increased with a slope of 1%/µm at distances between 50 and 300 µm from the soma. Sodium channels were not included. HCN channels (hyperpolarization-activated cyclic nucleotide-gated cation channel) were included as modeled in Migliore et al.^63^. The density was set to 0.2 mS/cm^2^ in soma and basal dendrites, and increased in the apical dendrites linearly with 3%/µm until a distance of 500 µm from the soma^63^. The reversal of the H current was –30 mV. And the reversal of the leak current was set to –90 mV. Under these conditions, the resting membrane potential was close to −70 mV (experiment: –67.2 ± 0.4 mV, *n* = 25), the apparent input resistance of the cell was 95.4 MΩ (exp.: 90.3 ± 4.5 MΩ) measured by small positive current injections into the soma, and a negative current step of –100 pA produced an I_h_-dependent sag of 2.1 mV (exp.: 2.4 ± 0.2 mV). If HCN channels were removed, the apparent input resistance increased to 219.4 MΩ (exp.: 217.8 ± 23.5 MΩ, *n* = 11) and the membrane time constant measured as the decay of the membrane potential from a small depolarizing current step was 43.7 ms (exp.: 44.3 ± 2.7 ms, *n* = 11). The spatial distribution of glutamatergic and GABAergic synaptic inputs was implemented as described in Bloss et al.^35^. The decay *τ* of the AMPA conductance was set to 3 ms.

In Fig. 8, increasing numbers of randomly chosen glutamate synapses together with 10 fixed nonlinear GABA synapses (~3.5% of total 280 GABAergic synapses in tuft dendrites) were activated in a short burst stimulation (3@200 Hz) to induce local NMDAR-mediated dendritic spikes in tuft dendrites. Based on Maccaferri et al.^17^, each GABA synapse had a total synaptic weight of 1 nS, generating a peak conductance of 0.4 nS at −70 mV, similar to what was reported for unitary SOM-interneuron pyramidal-cell synapses. The release probability was set to 1 due to the remarkably low failure rate of inputs from SOM+ OLM interneurons^17^.

For glutamate synapses, the release probability was assumed to be 0.1, so that the activation of 150 synapses (~10% of 1464) yielded 15 active synapses on the first pulse. The release probability was increased by a factor of 1.5 and 1.8 on the subsequent stimuli according to experimental data (see above). Short-term synaptic plasticity (STP) was implemented by the introduction of separate Network Connection objects (NetCon) for each stimulus and synapse. This allowed the independent activation of any number of synapses by the three stimulations represented by individual NetStim objects. During each round of the simulation, the number of potentially active glutamate synapses was increased by 150 synapses, i.e., by 15 additionally activated inputs during the first pulse of each subsequent burst. The following modifications of our nonlinear GABA synapse model were tested: for linear inhibition (Fig. 8d), the voltage-dependent rectification was turned off and the synaptic weight was fixed at 40% of 1 nS; for inputs with kinetics typical for PV+ basket- cell inputs (Fig. 8e), rise and decay *τ* of the rectifying and linear conductance were set to 0.2 ms and 6 ms, respectively, and the total synaptic weight was increased from 1 nS to 4.5 nS (=0.2 nS/0.4 + 0.8 nS/0.2) to achieve the same integral PSC (i.e., charge transfer) as for the slow nonlinear inhibition.

To approximately model the experimental situation with simultaneous stimulation in SR and SLM (Supplementary Fig. 8, Fig. 2d), inputs were distributed over the whole apical dendritic tree and activated during a burst stimulation of 5 stimuli @ 50 Hz. All dendritic GABAergic synapses were modeled as nonlinear GABA synapses, similar to what was described above. The synapses consisted of a 20% linear and an 80% rectifying component with *V*_slope_ = 4 mV and a half-maximal activation at *V*_50_ = –60 mV.

About 34% of all GABAergic synapses were randomly selected and activated (95 in SLM and 50 in SR). Synaptic weight of all dendritic GABAergic synapses was set to 0.7 nS and the initial release probability was set to 0.5. As GABAergic synapses located throughout the dendritic tree are a complex mixture formed by many different types of interneurons, we tried to experimentally determine the average STP during brief-burst stimulation (5@50 Hz). In SR, the relative short-term depression of IPSCs during the 2nd, 3rd, 4th, and 5th pulse relative to control was 0.7 ± 0.1, 0.8 ± 0.2, 0.7 ± 0.1, and 0.7 ± 0.1 (*n* = 11), respectively. Similar results were obtained with SLM stimulation (0.6 ± 0.1, 0.5 ± 0.1, 0.4 ± 0.1, and 0.4 ± 0.1, *n* = 16). Accordingly, the release probability was scaled during brief-burst stimulation by 0.7 in SR and by 0.6, 0.5, 0.4, and 0.4 in SLM. In addition to the described conductances, this model included a slow GABA_B_ conductance that was taken with small modifications from Poirazi et al.^64^. Each GABA_A_ synapse had directly next to it a GABA_B_ partner and the maximal GABA_B_ conductance was set to 0.4 nS to obtain GABA_B_-mediated IPSPs similar to what we have observed under comparable experimental conditions (data not shown).

Increasing numbers of glutamatergic synapses were activated starting with 200 synapses in SR (~3% of about 6000) and 50 in SLM (~3.3% of 1464). Release probability was set to 0.1 and scaled with the experimentally determined STP of 1, 1.5, 1.8, 1.9, and 2.0. During each round of the simulation, the number of potentially active glutamate synapses was increased by 250 additional synapses, i.e., by 25 activated inputs at the first pulse. In round 3 (75 glutamatergic inputs), there was an E/I-conductance ratio of ~1:2 for inputs onto dendrites within SR (generated by 60 glutamatergic and 25 GABAergic inputs), similar to what we have observed in voltage-clamp experiments with SR stimulation (unpublished data). This number of synapses resulted in a peak depolarization of ~13 mV measured at the soma that was very similar to current-clamp recordings of burst PSPs in Fig. 2d.

To test for the potential contribution of tonic inhibition (TI) to the effect of α5-NAM on PSP bursts (Supplementary Fig. 9), we included the outward-rectifying tonic conductance described in Pavlov et al.^38^. The TI conductance density increased with a slope of 3%/µm at distances between 50 and 300 µm from the soma. The initial TI conductance density was adjusted in current-clamp mode with IH turned off to obtain a change in the apparent resting conductance (the inverse of *R*_in_) after 50% reduction of TI comparable to the effect of the α5-NAM in experiments in the presence of ZD. A density of 0.1 mS/cm^2^ fulfilled this requirement. This lowered the somatic *R*_in_ from 219.1 MΩ to 192.9 MΩ (i.e., tonic conductance of 0.62 nS). Blocking half of this conductance increased the *R*_in_ to 204.9 MΩ, i.e., a change in resting conductance of 0.30 nS comparable to experimental observation in voltage and current clamp (Supplementary Figures 2G, 3A). In subsequent steps, the TI conductance density was increased by factors of 10 and 100 to simulate unrealistically high contributions of TI to the resting conductance.

### Code availability

The code for the simulations in NEURON will be deposited in ModelDB: https://senselab.med.yale.edu/modeldb/

## Electronic supplementary material

Supplementary Information

## Data Availability

The data that support the findings in this study are available from the corresponding author upon reasonable request.

## References

[1] Gentet LJ (2012). Unique functional properties of somatostatin-expressing GABAergic neurons in mouse barrel cortex. Nat. Neurosci..

[2] Wilson NR, Runyan CA, Wang FL, Sur M (2012). Division and subtraction by distinct cortical inhibitory networks in vivo. Nature.

[3] Lee S, Kruglikov I, Huang ZJ, Fishell G, Rudy B (2013). A disinhibitory circuit mediates motor integration in the somatosensory cortex. Nat. Neurosci..

[4] Cichon J, Gan WB (2015). Branch-specific dendritic Ca^2+^ spikes cause persistent synaptic plasticity. Nature.

[5] Cichon J, Blanck TJJ, Gan WB, Yang G (2017). Activation of cortical somatostatin interneurons prevents the development of neuropathic pain. Nat. Neurosci..

[6] Royer S (2012). Control of timing, rate and bursts of hippocampal place cells by dendritic and somatic inhibition. Nat. Neurosci..

[7] Lovett-Barron M (2012). Regulation of neuronal input transformations by tunable dendritic inhibition. Nat. Neurosci..

[8] Lovett-Barron M (2014). Dendritic inhibition in the hippocampus supports fear learning. Science.

[9] Takahashi H, Magee JC (2009). Pathway interactions and synaptic plasticity in the dendritic tuft regions of CA1 pyramidal neurons. Neuron.

[10] Bittner KC (2015). Conjunctive input processing drives feature selectivity in hippocampal CA1 neurons. Nat. Neurosci..

[11] Klausberger T, Somogyi P (2008). Neuronal diversity and temporal dynamics: the unity of hippocampal circuit operations. Science.

[12] Somogyi P, Katona L, Klausberger T, Lasztóczi B, Viney TJ (2013). Temporal redistribution of inhibition over neuronal subcellular domains underlies state-dependent rhythmic change of excitability in the hippocampus. Philos Trans. R. Soc. Lond. B Biol. Sci..

[13] Bezaire MJ, Raikov I, Burk K, Vyas D, Soltesz I (2016). Interneuronal mechanisms of hippocampal theta oscillations in a full-scale model of the rodent CA1 circuit. eLife.

[14] Jonas P, Bischofberger J, Fricker D, Miles R (2004). Interneuron Diversity series: fast in, fast out--temporal and spatial signal processing in hippocampal interneurons. Trends Neurosci..

[15] Bartos M, Vida I, Jonas P (2007). Synaptic mechanisms of synchronized gamma oscillations in inhibitory interneuron networks. Nat. Rev. Neurosci..

[16] Hu H, Gan J, Jonas P (2014). Interneurons. Fast-spiking, parvalbumin^+^ GABAergic interneurons: from cellular design to microcircuit function. Science.

[17] Maccaferri G, Roberts JD, Szucs P, Cottingham CA, Somogyi P (2000). Cell surface domain specific postsynaptic currents evoked by identified GABAergic neurones in rat hippocampus in vitro. J. Physiol..

[18] Collinson N (2002). Enhanced learning and memory and altered GABAergic synaptic transmission in mice lacking the alpha 5 subunit of the GABAA receptor. J. Neurosci..

[19] Zarnowska ED, Keist R, Rudolph U, Pearce RA (2009). GABAA receptor alpha5 subunits contribute to GABAA, slow synaptic inhibition in mouse hippocampus. J. Neurophysiol..

[20] Vargas-Caballero M, Martin LJ, Salter MW, Orser BA, Paulsen O (2010). α5 subunit-containing GABAA receptors mediate a slowly decaying inhibitory synaptic current in CA1 pyramidal neurons following Schaffer collateral activation. Neuropharmacol.

[21] Capogna M, Pearce RA (2011). GABAA slow: causes and consequences. Trends Neurosci..

[22] Farrant M, Nusser Z (2005). Variations on an inhibitory theme: phasic and tonic activation of GABAA receptors. Nat. Rev. Neurosci..

[23] Serwanski DR (2006). Synaptic and nonsynaptic localization of GABAA receptors containing the alpha5 subunit in the rat brain. J. Comp. Neurol..

[24] Möhler H, Rudolph U (2017). Disinhibition, an emerging pharmacology of learning and memory. F1000Res..

[25] Fritschy JM, Möhler H (1995). GABAA-receptor heterogeneity in the adult rat brain: differential regional and cellular distribution of seven major subunits. J. Comp. Neurol..

[26] Prenosil GA (2006). Specific subtypes of GABAA receptors mediate phasic and tonic forms of inhibition in hippocampal pyramidal neurons. J. Neurophysiol..

[27] Pirker S, Schwarzer C, Wieselthaler A, Sieghart W, Sperk G (2000). GABAA receptors: immunocytochemical distribution of 13 subunits in the adult rat brain. Neurosci.

[28] Ballard TM (2009). RO4938581, a novel cognitive enhancer acting at GABAA alpha5 subunit-containing receptors. Psychopharmacol.

[29] Martínez-Cué C (2013). Reducing GABAA α5 receptor-mediated inhibition rescues functional and neuromorphological deficits in a mouse model of down syndrome. J. Neurosci..

[30] Caraiscos VB (2004). Tonic inhibition in mouse hippocampal CA1 pyramidal neurons is mediated by alpha5 subunit-containing gamma-aminobutyric acid type A receptors. Proc. Natl Acad. Sci. USA.

[31] Brickley SG, Mody I (2012). Extrasynaptic GABAA receptors: their function in the CNS and implications for disease. Neuron.

[32] Kapur A, Lytton WW, Ketchum KL, Haberly LB (1997). Regulation of the NMDA component of EPSPs by different components of postsynaptic GABAergic inhibition: computer simulation analysis in piriform cortex. J. Neurophysiol..

[33] Quirk K (1996). McKernan, R.M. [3H]L-655,708, a novel ligand selective for the benzodiazepine site of GABAA receptors which contain the alpha 5 subunit. Neuropharmacol.

[34] Armstrong C, Krook-Magnuson E, Soltesz I (2012). Neurogliaform and Ivy cells: a major family of nNOS expressing GABAergic neurons. Front. Neural Circuits.

[35] Bloss EB (2016). Structured dendritic inhibition supports branch-selective integration in CA1 pyramidal cells. Neuron.

[36] Petreanu L, Huber D, Sobczyk A, Svoboda K (2007). Channelrhodopsin-2-assisted circuit mapping of long-range callosal projections. Nat. Neurosci..

[37] Petreanu L, Mao T, Sternson SM, Svoboda K (2009). The subcellular organization of neocortical excitatory connections. Nature.

[38] Pavlov I, Savtchenko LP, Kullmann DM, Semyanov A, Walker MC (2009). Outwardly rectifying tonically active GABAA receptors in pyramidal cells modulate neuronal offset, not gain. J. Neurosci..

[39] Ali AB, Thomson AM (2008). Synaptic alpha 5 subunit-containing GABAA receptors mediate IPSPs elicited by dendrite-preferring cells in rat neocortex. Cereb. Cortex.

[40] Brady ML, Jacob TC (2015). Synaptic localization of α5 GABAA receptors via gephyrin interaction regulates dendritic outgrowth and spine maturation. Dev. Neurobiol..

[41] Rudolph U, Knoflach F (2011). Beyond classical benzodiazepines: novel therapeutic potential of GABAA receptor subtypes. Nat. Rev. Drug Discov..

[42] Larkum ME, Nevian T, Sandler M, Polsky A, Schiller J (2009). Synaptic integration in tuft dendrites of layer 5 pyramidal neurons: a new unifying principle. Science.

[43] Major G, Larkum ME, Schiller J (2013). Active properties of neocortical pyramidal neuron dendrites. Annu. Rev. Neurosci..

[44] Palmer LM (2014). NMDA spikes enhance action potential generation during sensory input. Nat. Neurosci..

[45] Kapfer C, Glickfeld LL, Atallah BV, Scanziani M (2007). Supralinear increase of recurrent inhibition during sparse activity in the somatosensory cortex. Nat. Neurosci..

[46] Silberberg G, Markram H (2007). Disynaptic inhibition between neocortical pyramidal cells mediated by Martinotti cells. Neuron.

[47] Pi HJ (2013). Cortical interneurons that specialize in disinhibitory control. Nature.

[48] Braat S, Kooy RF (2015). The GABAA receptor as a therapeutic target for neurodevelopmental disorders. Neuron.

[49] Soh MS, Lynch JW (2015). Selective modulators of α5-containing GABAA receptors and their therapeutic significance. Curr. Drug Targets.

[50] Fischell J, Van Dyke AM, Kvarta MD, LeGates TA, Thompson SM (2015). Rapid antidepressant action and restoration of excitatory synaptic strength after chronic stress by negative modulators of alpha5-containing GABAA receptors. Neuropsychopharmacol.

[51] Zanos P (2017). A negative allosteric modulator for α5 subunit-containing GABA receptors exerts a rapid and persistent antidepressant-like action without the side effects of the NMDA receptor antagonist ketamine in mice. eNeuro.

[52] Mendez MA (2013). The brain GABA-benzodiazepine receptor alpha-5 subtype in autism spectrum disorder: a pilot [(11)C]Ro15-4513 positron emission tomography study. Neuropharmacol.

[53] Robertson CE, Ratai EM, Kanwisher N (2016). Reduced GABAergic action in the autistic brain. Curr. Biol..

[54] Martin LJ (2010). Alpha5-GABAA receptor activity sets the threshold for long-term potentiation and constrains hippocampus-dependent memory. J. Neurosci..

[55] Zurek AA (2016). α5GABAA receptor deficiency causes autism-like behaviors. Ann. Clin. Transl. Neurol..

[56] Geiger JRP (2002). Patch-clamp recording in brain slices with improved slicer technology. Pflügers Arch..

[57] Bischofberger J, Engel D, Li L, Geiger JRP, Jonas P (2006). Patch-clamp recording from mossy fiber terminals in hippocampal slices. Nat. Protoc..

[58] Glykys J, Mody I (2007). The main source of ambient GABA responsible for tonic inhibition in the mouse hippocampus. J. Physiol..

[59] Guzman SJ, Schlögl A, Schmidt-Hieber C (2014). Stimfit: quantifying electrophysiological data with Python. Front. Neuroinform..

[60] Hines ML, Carnevale NT (1997). The neuron simulation environment. Neural Comput..

[61] Jahr CE, Stevens CF (1990). Voltage dependence of NMDA-activated macroscopic conductances predicted by single-channel kinetics. J. Neurosci..

[62] Kirizs T, Kerti-Szigeti K, Lorincz A, Nusser Z (2014). Distinct axo-somato-dendritic distributions of three potassium channels in CA1 hippocampal pyramidal cells. European. J. Neurosci..

[63] Migliore M, Messineo L, Ferrante M (2004). Dendritic Ih selectively blocks temporal summation of unsynchronized distal inputs in CA1 pyramidal neurons. J. Comput. Neurosci..

[64] Poirazi P, Brannon T, Mel BW (2003). Arithmetic of subthreshold synaptic summation in a model CA1 pyramidal cell. Neuron.

