# Supplementary material for: Dendrite-targeting interneurons control synaptic NMDA-receptor activation via nonlinear α5-GABAA receptors
Source: Nat Commun. 2018 Sep 3;9:3576. doi: 10.1038/s41467-018-06004-8 (PMC6120902; doi:10.1038/s41467-018-06004-8)
Supplement: Supplementary file 1 — Supplementary Information [file 41467_2018_6004_MOESM1_ESM.pdf]

Supplementary Information for:

Dendrite-targeting interneurons control synaptic NMDA- receptor activation via nonlinear α5-GABA$_A$ receptors

Jan M. Schulz[1], Frederic Knoflach[2], Maria-Clemencia Hernandez[2], and Josef Bischofberger[1]

Table of contents:

Supplementary Fig. 1-9

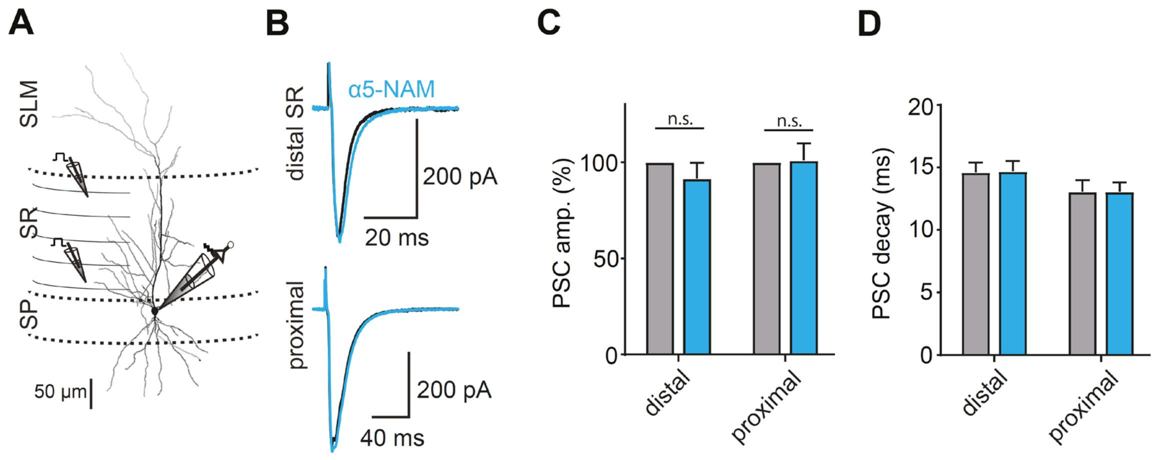

Supplementary Figure 1. α5-NAM RO4938581 does not affect AMPAR-mediated EPSCs.

A) Experimental design for local stimulation of AMPAR-mediated EPSCs in the outer third and inner third of the SR. AMPAR-EPSCs were recorded in the presence of 100 µM picrotoxin and 25 µM AP5 using a Cs-gluconate based solution. **B**) Mean AMPAR-EPSC in control (black) and after addition of the α5-NAM RO4938581 (1 µM). **C**) Group means of the normalized EPSC amplitude were unchanged after α5-NAM independent of stimulation location (distal SR: P=0.61; proximal: P=0.29; paired t-test, n=6). **D**) Group means of decays show that EPSCs evoked at distal synapses had only somewhat slower decays (14.6±0.8 ms, n=6) than EPSCs evoked at proximal synapses (13.1±0.9 ms, n=6). Recording temperature T≈22°C.

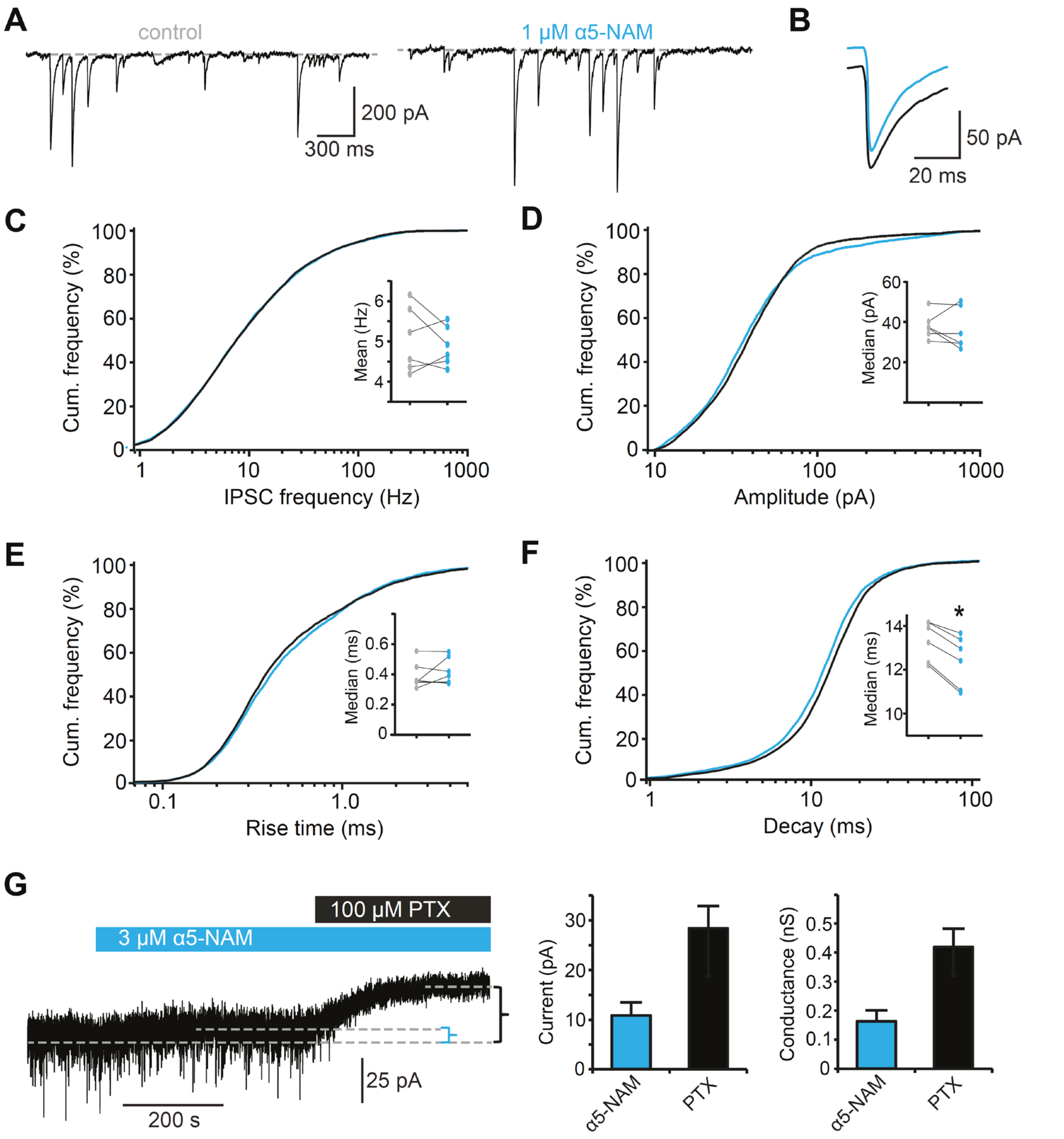

Supplementary Figure 2. α5-GABA$_A$Rs contribute to tonic inhibition in CA1 pyramidal neurons but not to amplitude and frequency of spontaneous IPSCs.

A) Example traces of spontaneous IPSCs (sIPSCs) before and after addition of the α5-NAM, recorded at room temperature. Dashed lines indicate the α5-NAM-induced change in tonic median membrane current from -284 pA to -273 pA. **B**) Mean waveform of sIPSCs in control (black) and after addition of the α5-NAM. **C-E**), Cumulative distributions show no significant effect of the α5-NAM on sIPSC frequency (C), amplitude (D) or rise τ of IPSCs (E). **F**) By contrast, the decay time constant of sIPSCs was marginally, but significantly reduced (*P*<0.001; paired t-test, *n*=6). **G**) Voltage-clamp recording showing the reduction in holding current after addition of the α5-NAM in the presence of 5 µM GABA. Picrotoxin (PTX) blocked GABA-dependent tonic currents completely. Mean reduction of tonic current by the α5-NAM was 37.7 ± 5.5% (*n*=11) of the PTX-sensitive current.

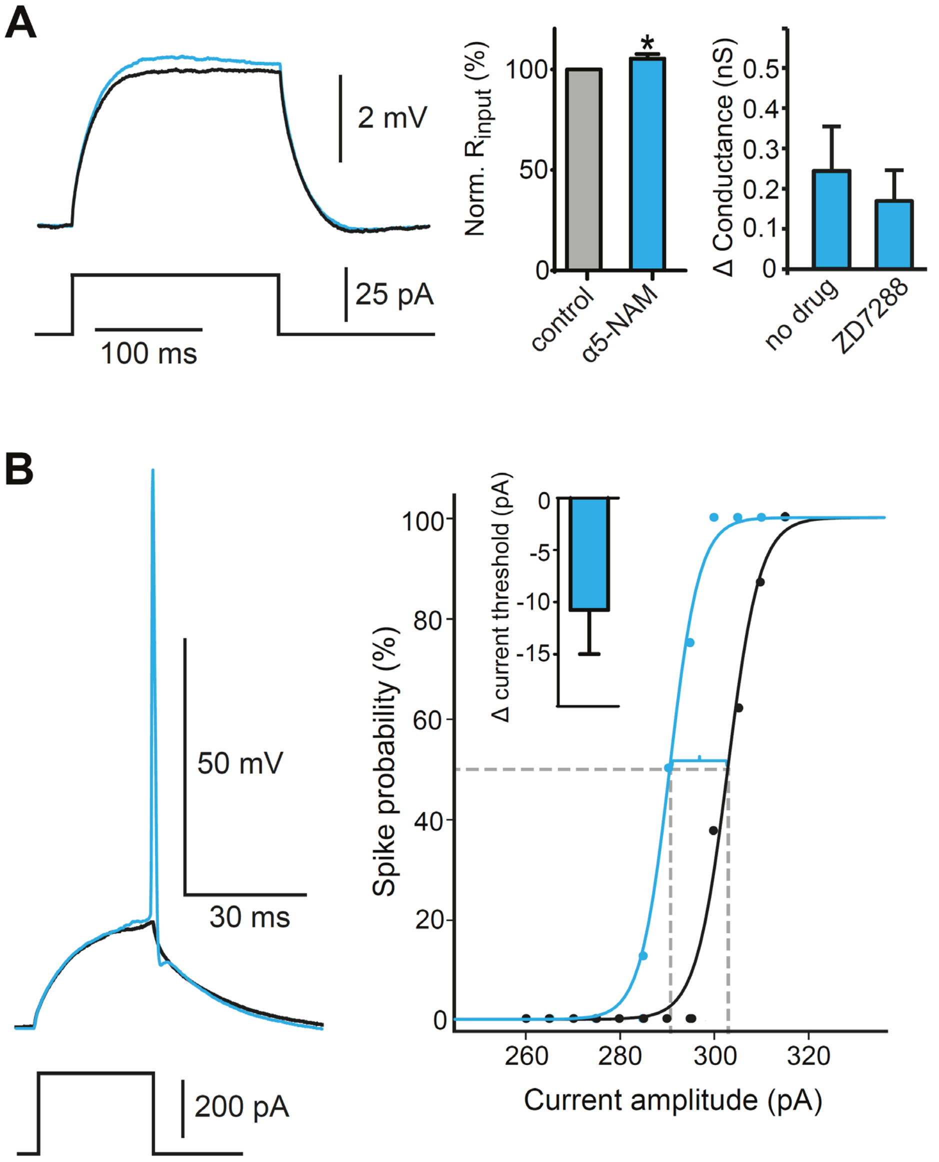

Supplementary Figure 3. Application of the α5-NAM moderately increases cellular excitability.

A) Membrane potential response to a 25 pA current step before (black) and after the application of the α5-NAM (1 µM; blue) in the absence of added GABA. The right panel shows that the mean input resistance was moderately increased by 3.9 ± 1.5% (*P*<0.01; Wilcoxon Signed Rank test, *n*=18) after the addition of the α5-NAM. The calculated underlying conductance change was about 0.2 nS and similar in experiments with and without the HCN channel blocker ZD7288. **B**) Left, example membrane potential response to a short current step that was just subthreshold in control condition (30 ms, 290 pA; black). After the application of the α5-NAM (blue), the neuron responded with a spike in 50% of cases. Right, the spike probability is shown in dependence of the current step amplitude. Sigmoidal fits are indicated. The inset shows the average decrease in the current amplitude necessary to evoke APs in 50% of trials, which corresponds to 4.6 ±1.7% (*P*<0.05; paired t-test, *n*=6) of the control current amplitude. These results show that α5-GABAR-mediated tonic inhibition has a small but significant influence on cellular excitability.

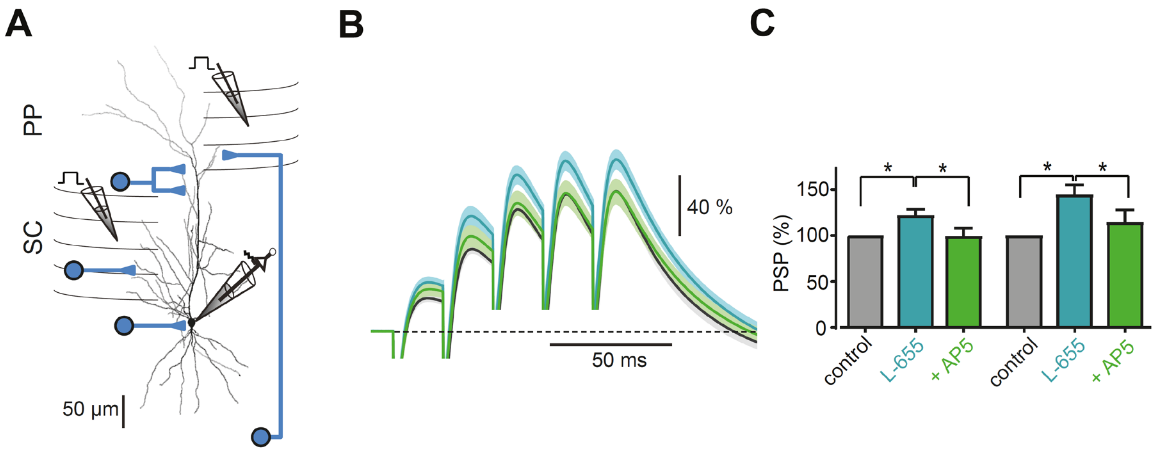

Supplementary Figure 4. α5-IA L-655,708 increases NMDAR activation during brief burst PSPs.

A) Experimental design for local stimulation of glutamatergic inputs from Schaffer Collaterals (S.C.) and Perforant Path (P.P.) as well as associated GABAergic inputs. **B**) Enhanced contribution of NMDARs after application of the α5-IA L-655,708 (50 nM). The Grand Means with SEM (lighter shades) of 10 experiments are shown. Each mean was normalized to the maximal voltage deflection in the control condition (grey). Application of L-655 increased the burst PSP (blue), the addition of AP5 (50 µM, green) completely reversed this effect. **C**) Group means of the amplitude and integral of the burst PSP. Statistical significant differences are indicated ($P<0.05$; paired t-test, $n=10$). Recording temperature T≈33°C.

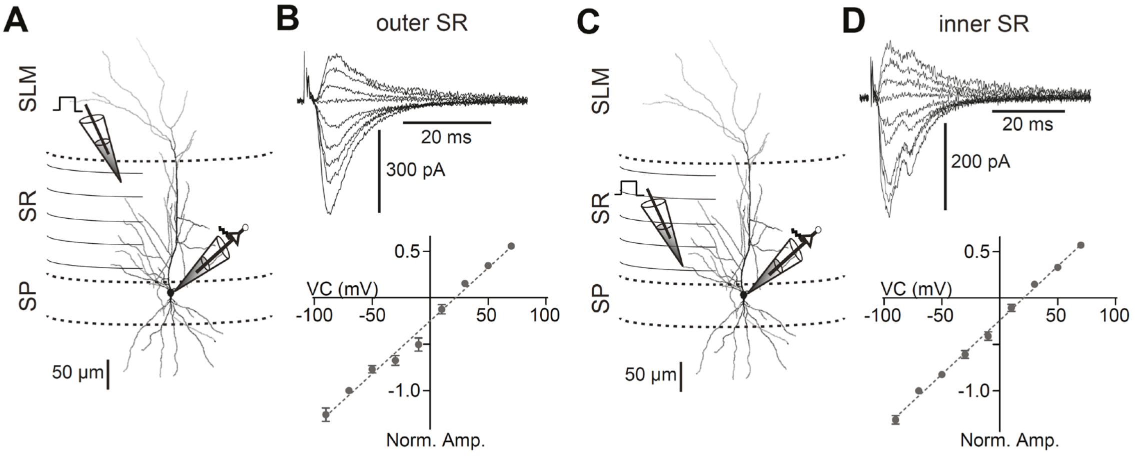

Supplementary Figure 5. No distance-dependent rectification of AMPAR-mediated EPSCs.

A) Experimental design for local stimulation of EPSCs in the outer third of SR. **B**) AMPAR-mediated EPSCs evoked at the border between SLM and SR were recorded at different membrane potentials in the presence of 10 µM gabazine and 25 µM AP5 using a Cs-gluconate based solution. Bottom, group data (*n*=6) show the mean EPSC amplitudes depending on the holding potential. The dashed line represents a linear fit. Note that there are no signs for a voltage-dependent deviation from the linear fit. **C**+**D**) The same as A and B for proximally evoked EPSCs in the inner third of SR. All recordings were performed with whole-cell Rs-compensation (80%). Recording temperature T≈33°C.

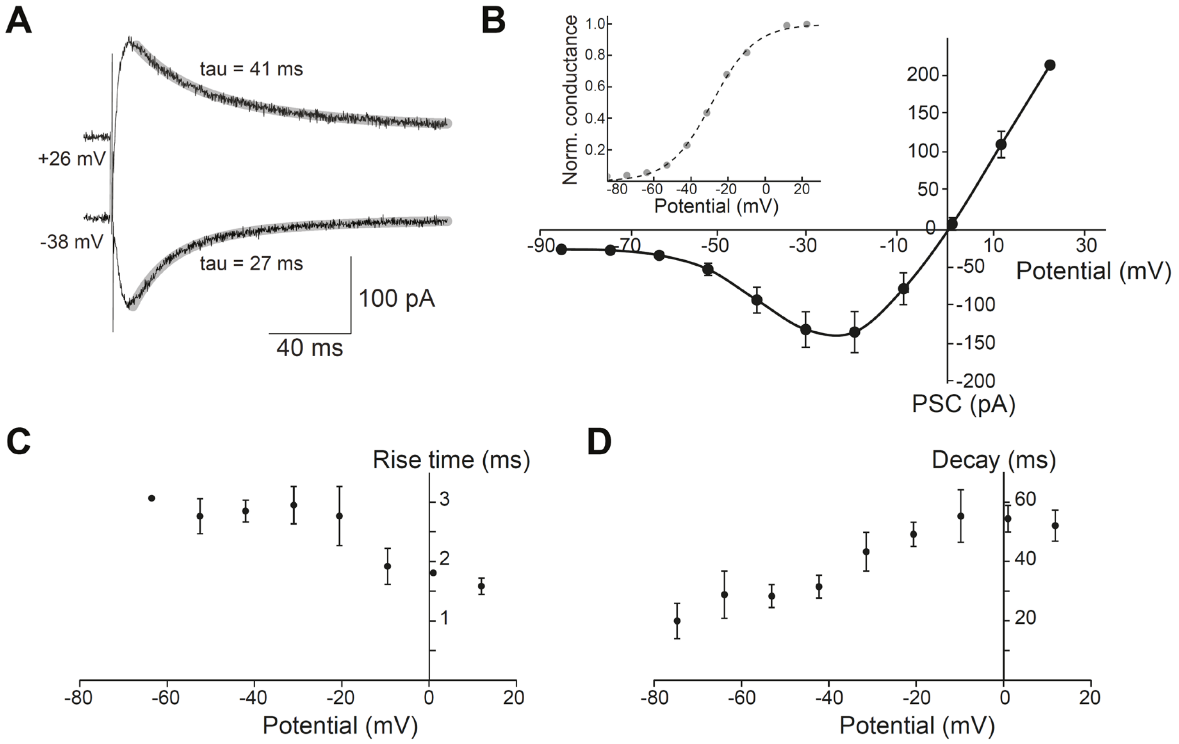

Supplementary Figure 6. Voltage-dependent conductance profile and kinetics of synaptic NMDARs recorded in CA1 pyramidal neurons.

A) Representative outward and inward NMDAR-EPSCs. The holding potential is indicated. A monoexponential fit to the decay phase is shown in grey. Synaptic inputs were stimulated in SR or SO close to the soma in the presence of 20 µM CNQX and 10 µM gabazine. **B**) The relationship between mean PSC amplitude and membrane potential. Inset shows the calculated voltage-dependent conductance and fitted sigmoidal (dashed line). **C**) Voltage-dependence of the rise and (**D**) decay τ. Recording temperature T≈33°C.

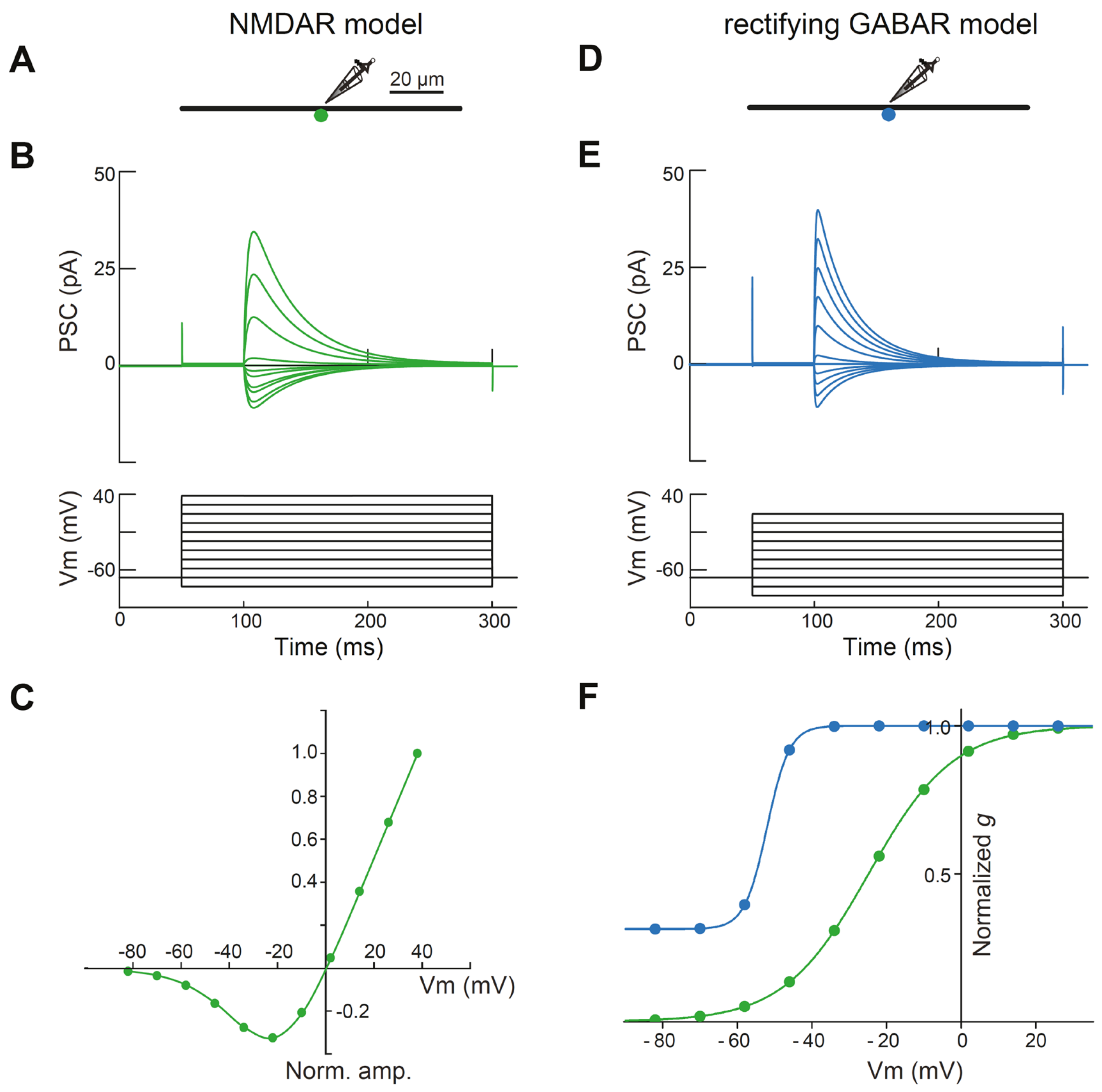

Supplementary Figure 7. Computational models of NMDAR and nonlinear outward-rectifying GABAR.

A) View of the dendritic branch model. NMDAR and recording location are indicated. **B**) Overlay of voltage-clamp simulations of NMDAR-EPSCs at different holding potentials. Voltage steps are shown in the lower panel. **C**) Current-voltage relationship for the NMDAR model. **D-E**) $GABA_AR$-mediated PSCs simulated at different voltages, modeling the sum of a 20% fast and linear component and an 80% outward-rectifying component (see methods) as used for simulations in Fig. 6-8. Voltage steps are shown in the lower panel. **F**) Voltage-dependence of the normalized NMDAR-mediated (green) and nonlinear GABAR-mediated conductance (blue) superimposed.

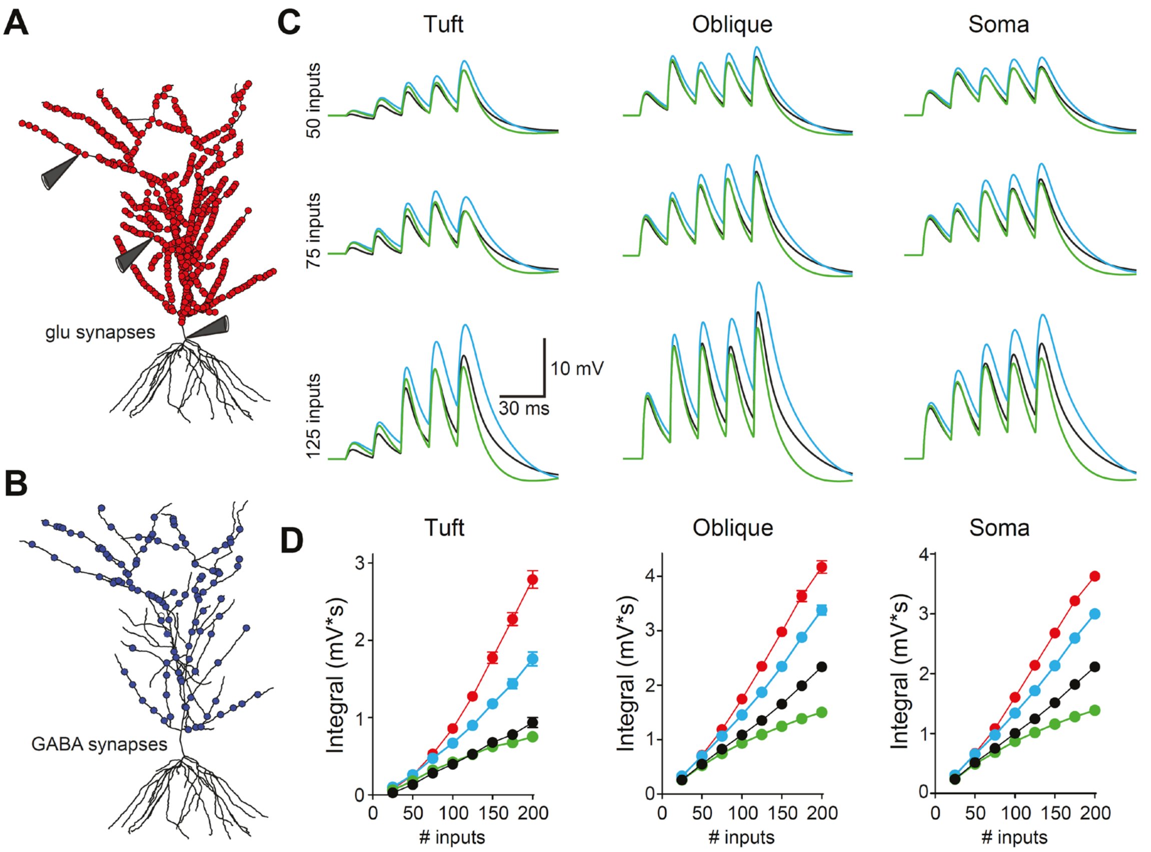

Supplementary Figure 8. Effects of non-linear GABAergic inhibition on NMDAR recruitment in a CA1-pyramidal cell model during stimulation of Schaffer-Collateral and Perforant-Path inputs.

A) View of the pyramidal model. Stimulated glutamatergic synapses (red circles) and recording locations are indicated. **B**) Location of stimulated GABAergic synapses (blue). **C**) Membrane potential responses in different compartments of the model neuron to burst stimulation (5@50 Hz) of increasing numbers of glutamatergic synapses (50, 75 and 125) are shown for three different conditions: black, control, i.e. nonlinear slow GABARs, NMDAR and AMPAR are included; blue, 50% reduced outward-rectifying component of GABARs mimicking the application of an α5-NAM; green, removal of NMDARs mimicking the addition of AP5 in physiological experiments, while the outward-rectifying component of GABARs remains reduced. The number of activated GABA synapses stayed constant across simulations. Note the increase of the voltage envelope by the reduction of the outward-rectification in GABARs and the subsequent return close to control levels by the removal of NMDARs reminiscent of the experimental observations reported in Fig. 4. **D**) The dependence of the burst integral in different neuronal compartments on glutamatergic input number. For the soma, each point is the mean of 4 simulations with randomized glutamatergic and GABAergic synapse locations. For tuft and oblique dendrites, each point is the mean of 4 simulations across three different dendritic branches. The SEM is indicated. The following conditions are shown: red: linear GABARs; black, nonlinear GABARs; blue, reduced nonlinear GABARs; green, no NMDARs and reduced nonlinear GABARs.

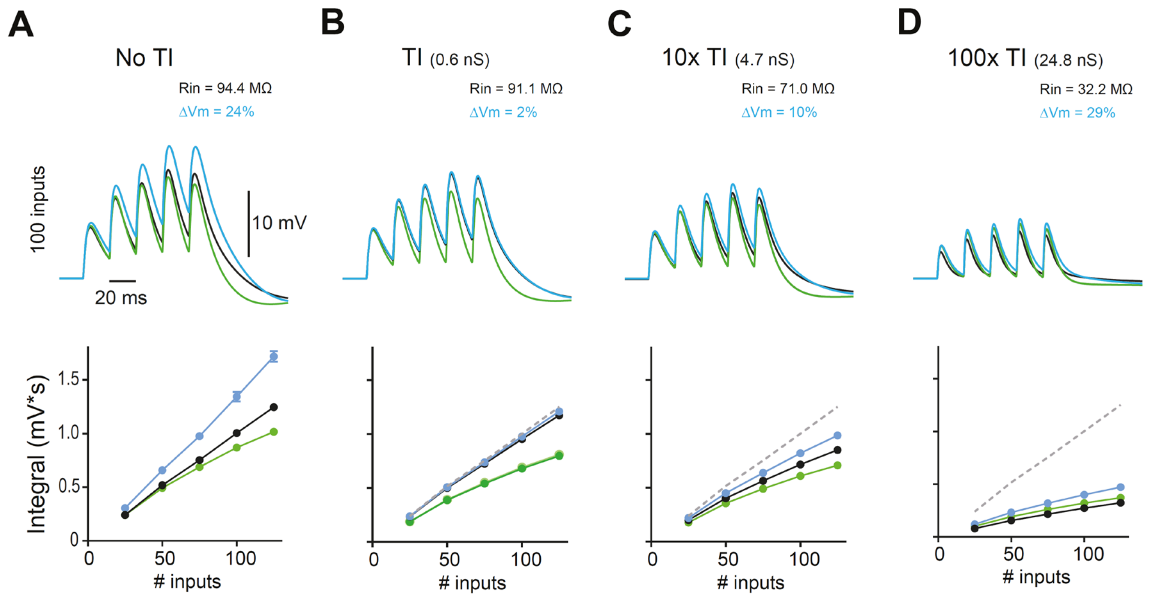

Supplementary Figure 9. Small contribution of tonic inhibition (TI) to α5-GABARs mediated control of NMDAR recruitment in a CA1 pyramidal cell model.

A) Somatic membrane potential responses to burst stimulation (5@50 Hz) in the same model as in Fig. S8 are shown for three different conditions: black, control including nonlinear slow GABARs; blue, 50% reduction of outward-rectifying component of GABARs mimicking the application of the α5-NAM; green, subsequent removal of NMDARs mimicking the addition of AP5. Input resistance (R_{in}) and the increase of the burst PSP amplitude (ΔVm) after the application of the α5-NAM are indicated. Bottom, the dependence of the burst PSP integral on synaptic input numbers. Each point is the mean of 5 simulations with randomized glutamatergic and GABAergic synapse locations. **B**) The same model including tonically active α5-$GABA_A$Rs (TI) in apical dendrites. The density was set to 0.1 mS/cm^2 close to soma and increased linearly with 3%/µm until a distance of 300 µm from the soma (see Methods for details). The apparent tonic conductance at the soma was estimated from Rin measurements (*in silico*) as indicated and reproduced experimentally measured TI. Reducing TI by 50% while leaving phasic synaptic inhibition unchanged has minimal effects on the voltage integral (blue). Increasing the TI conductance density by a factor of 10 (**C**) or 100 (**D**) increases the α5-NAM-induced effect on the PSP integral, but also reduces R_{in} dramatically.
